# Fabclavine diversity in *Xenorhabdus* bacteria

**DOI:** 10.3762/bjoc.16.84

**Published:** 2020-05-07

**Authors:** Sebastian L Wenski, Harun Cimen, Natalie Berghaus, Sebastian W Fuchs, Selcuk Hazir, Helge B Bode

**Affiliations:** 1 Molekulare Biotechnologie, Fachbereich Biowissenschaften, Goethe Universität Frankfurt, Max-von-Laue-Str. 9, 60438 Frankfurt, Germany; 2 Adnan Menderes University, Faculty of Arts and Sciences, Department of Biology, 09010 Aydin, Turkey; 3 Buchmann Institute for Molecular Life Sciences (BMLS), Goethe Universität Frankfurt, Max-von-Laue-Str. 15, 60438 Frankfurt, Germany; 4 Senckenberg Gesellschaft für Naturforschung, Senckenberganlage 25, 60325 Frankfurt am Main, Germany

**Keywords:** antibiotic, fabclavine, NRPS-PKS hybrid, secondary metabolite, *Xenorhabdus*

## Abstract

The global threat of multiresistant pathogens has to be answered by the development of novel antibiotics. Established antibiotic applications are often based on so-called secondary or specialized metabolites (SMs), identified in large screening approaches. To continue this successful strategy, new sources for bioactive compounds are required, such as the bacterial genera *Xenorhabdus* or *Photorhabdus*. In these strains, fabclavines are widely distributed SMs with a broad-spectrum bioactivity. Fabclavines are hybrid SMs derived from nonribosomal peptide synthetases (NRPS), polyunsaturated fatty acid (PUFA), and polyketide synthases (PKS). Selected *Xenorhabdus* and *Photorhabdus* mutant strains were generated applying a chemically inducible promoter in front of the suggested fabclavine (*fcl*) biosynthesis gene cluster (BGC), followed by the analysis of the occurring fabclavines. Subsequently, known and unknown derivatives were identified and confirmed by MALDI–MS and MALDI–MS^2^ experiments in combination with an optimized sample preparation. This led to a total number of 22 novel fabclavine derivatives in eight strains, increasing the overall number of fabclavines to 32. Together with the identification of fabclavines as major antibiotics in several entomopathogenic strains, our work lays the foundation for the rapid fabclavine identification and dereplication as the basis for future work of this widespread and bioactive SM class.

## Introduction

The constantly increasing threat of multiresistant pathogens requires the development of new antibiotics, as they are indispensable to maintain the state of health of our society [1]. Bacterial natural products, also called secondary or specialized metabolites (SM), such as daptomycin, vancomycin, or erythromycin, have already been shown to be potent antibiotics [2–4]. Consequently, research in the field of novel SMs with antimicrobial activity is vital to provide new avenues to new antiinfective drugs or lead compounds.

Beside traditional sources such as actinomycetes and myxobacteria, the genera *Photorhabdus* and *Xenorhabdus* are promising sources to discover new SMs since up to 6.5% of their overall genome sequence are associated with SM biosynthesis [5–6]. This includes antimicrobials like isopropylstilbene, xenocoumacins, amicoumacin, and several other SMs [7–11]. Naturally, *Photorhabdus* and *Xenorhabdus* are living in mutualistic symbiosis with nematodes of the genera *Steinernema* or *Heterorhabditis,* respectively [5,12]. Together, they infect and kill soil-living insects to use the cadaver as a food source and shelter [5]. After the infection of the insect by the nematode, the bacteria are released from the nematode gut into the insect hemocoel where they start producing a diversity of different natural products to suppress the immune response and to kill the insects, to defend the carcass against food competitors, and to trigger the development of the nematode [5,13].

The general interest on *Photorhabdus* and *Xenorhabdus* increased in recent years, not only because of their large number of SMs, but also due to their easy-to-handle cultivation under laboratory conditions in combination with the accessibility for genetic manipulations such as genomic integrations or deletions [14–17]. Furthermore, recently published studies focused on the possible application of *Photorhabdus* and *Xenorhabdus* as biological pest control agents with and without the corresponding nematodes [18–19].

**Figure 1:**
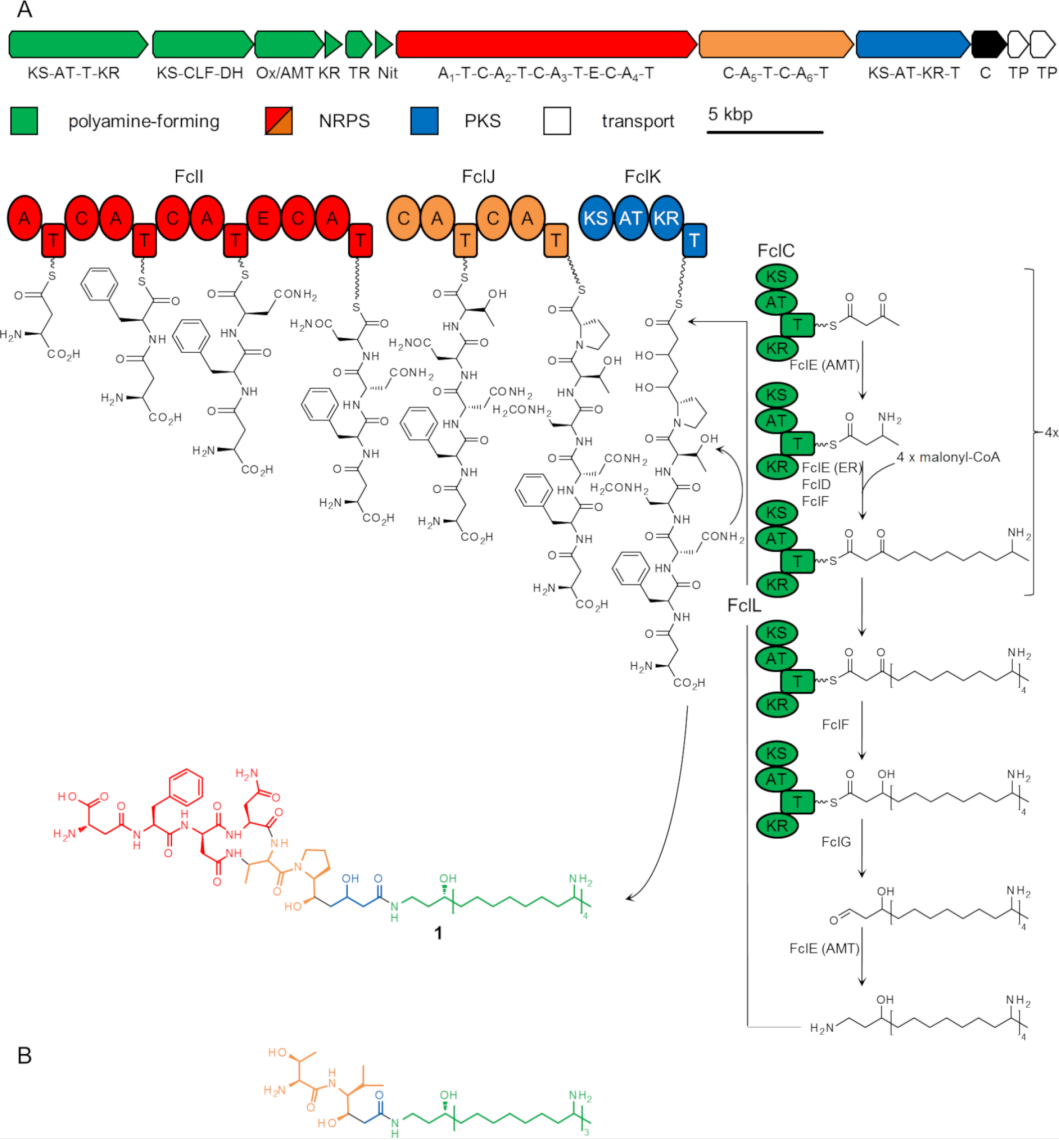
General biosynthesis of fabclavine Ia (**1**) in *X. budapestensis* (A) and representation of a shortened fabclavine derivative from *X. szentirmaii* (B), resulting from the alternative biosynthesis start at FclJ (the Figures were adapted and modified from [20] and [22]. KS: ketosynthase, AT: acyltransferase, T: thiolation domain, KR: ketoreductase, CLF: chain length factor domain, DH: dehydratase, Ox: 2-nitropropane dioxygenase (enoyl reductase), AMT: aminotransferase, TR: thioester reductase, Nit: nitrilase, A: adenylation, C: condensation, E: epimerization, TP: transport.

In 2014, the fabclavines were identified in *X. budapestensis* and *X. szentirmaii*, and a 50 kb biosynthesis gene cluster (BGC) was identified to be responsible for their formation (Figure 1) [20]. These compounds were of special interest because of their broad-spectrum bioactivity against Gram-positive and -negative bacteria, fungi, and protozoa [20–21]. Fabclavines are hexapeptide/polyketide hybrids derived from nonribosomal peptide synthetases (NRPS) and a polyketide synthase (PKS), which are connected to an unusual polyamine derived from polyunsaturated fatty acid (PUFA) synthases [20]. Beside full-length fabclavines, also shortened derivatives were identified. These are generated when the peptide biosynthesis starts directly with the second NRPS enzyme FclJ, which results in the formation of a dipeptide instead of the usual hexapeptide (Figure 1) [22]. Structurally related compounds are the (pre)zeamines described for *Serratia plymuthica* and *Dickeya zeae* [23–24]. They also exhibit broad-spectrum bioactivity, but their biosynthesis includes an additional processing step, executed by an acylpeptide hydrolase, which could not be detected in the fabclavine BGC [20,25].

To date, 10 full-length fabclavines could be identified, and the structure of fabclavine Ia (**1**) could be determined by NMR spectroscopy [20]. Furthermore, bioinformatic analysis of *Xenorhabdus* and *Photorhabdus* genomes revealed that the ability to produce fabclavines or related compounds might be widespread in these strains [22,26]. In order to analyze the associated fabclavine diversity, selected strains were analyzed both chemically and genomically, and mutants in putative *fcl* BGCs were generated. Thereby, a list of derivatives was obtained, which was further correlated to the potential fabclavine-producing, but genetically not accessible *X. innexi* strain. Finally, the bioactivity of the culture supernatants was analyzed, revealing that the fabclavines contribute largely to the overall bioactivity of *Xenorhabdus* when grown under laboratory conditions.

## Results

### Biosynthetic gene clusters for the fabclavine production are highly conserved

**Figure 2:**
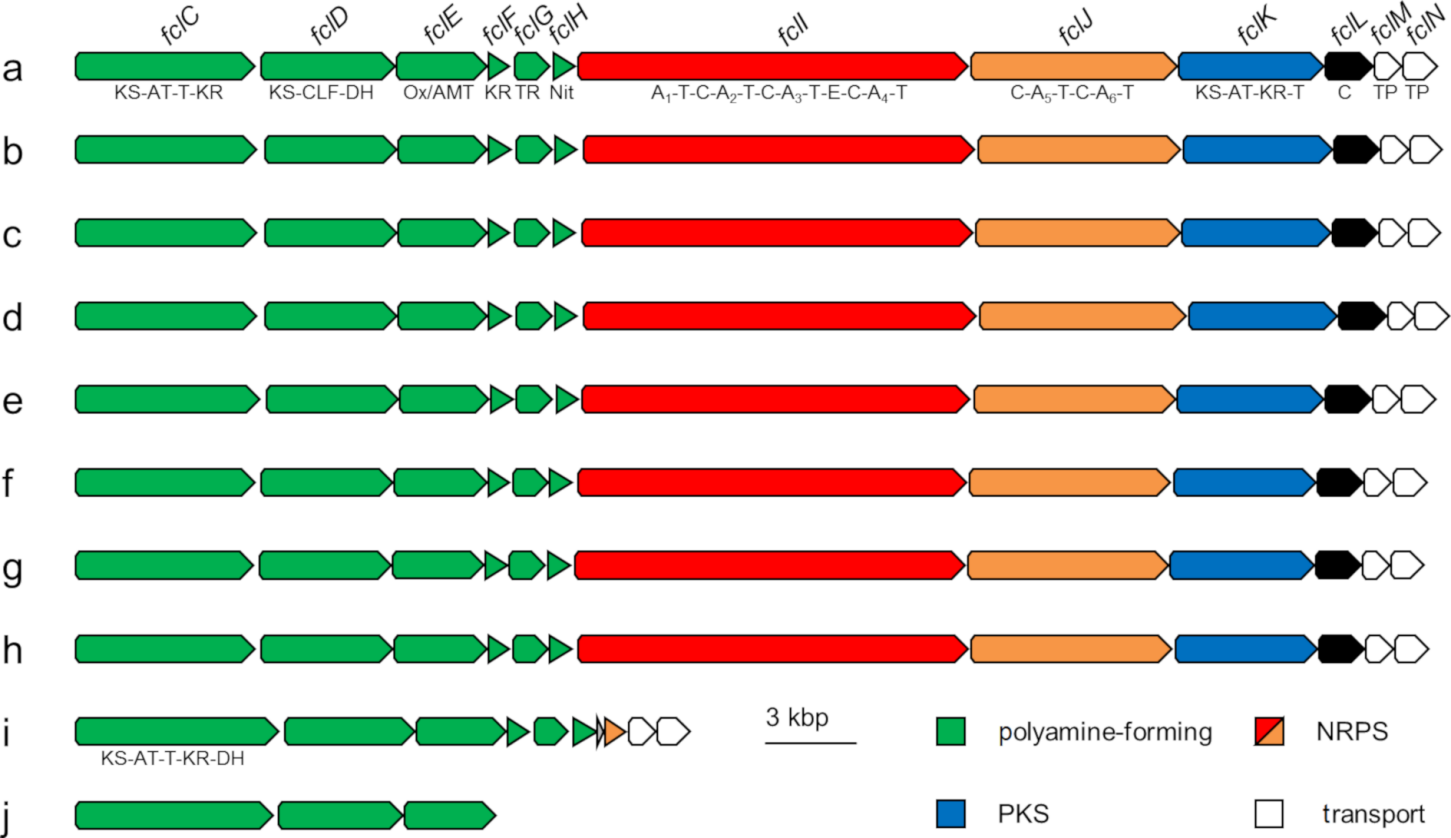
Comparison of the *fcl* BGCs in *Xenorhabdus* and *Photorhabdus* strains responsible for the fabclavine biosynthesis. a: *X. szentirmaii*, b: *X. budapestensis*, c: *X. cabanillasii*, d: *X. indica*, e: *X. hominickii*, f: *X. stockiae*, g: KK7.4, h: KJ12.1, i: *X. bovienii*, j: *P. temperata*. KS: ketosynthase, AT: acyltransferase, T: thiolation domain, KR: ketoreductase, CLF: chain length factor domain, DH: dehydratase, Ox: 2-nitropropane dioxygenase (enoyl reductase), AMT: aminotransferase, TR: thioester reductase, Nit: nitrilase, A: adenylation, C: condensation, E: epimerization, TP: transport.

During the screening for homologous *fcl* BGCs in *Xenorhabdus* and *Photorhabdus* strains, several candidate clusters were identified, which were conserved both in their BGC synteny as well as at the single protein level (Figure 2) [22].

The strain KK7.4 showed protein identities of ≥95% with *X. stockiae* and strain KJ12.1 (Figure S1, Supporting Information File 1). Similar identities could be observed for *X. budapestensis*, *X. cabanillasii*, and *X. indica* (≥91%, Table S3, Supporting Information File 1). Although both groups of strains also clustered together due to their close evolutionary relationship, the question was whether they would also produce the same fabclavine derivatives [26].

The BGC of *X. bovienii* encodes only the genes responsible for the polyamine biosynthesis as well as the transporter genes. A cryptic homologue of the NRPS *fclJ* in combination with the overall BGC structure suggested that the *fcl* BGC of *X. bovienii* originally also contained the NRPS-PKS-hybrid genes (Figure 2) [22]. In contrast, the BGC of *P. temperata* is reduced to only harbor the homologous genes of *fclC*, *fclD* and *fclE* (Figure 2) [22].

*X. innexi* also harbors a *fcl*-like BGC, with protein identities of 68–90% compared to *X. stockiae* (Figure S1, Supporting Information File 1). Nevertheless, *X. innexi* contains a *tonB*-homologue instead of the NUDIX-hydrolase *fclA* and an acyl-CoA-thioesterase instead of *fclM* and *fclN*, leading to the postulated compound Xenorhabdus lipoprotein toxin (Xlt, Figure S1, Supporting Information File 1) [27].

Furthermore, homologous BGCs can be found in *Serratia plymuthica* as well as in *Dickeya zeae* [20,25]. Like *Xenorhabdus* and *Photorhabdus,* these bacteria also belong to the order *Enterobacterales* and are producers of zeamines, which are structurally closely related to fabclavines and differ only in a postbiosynthetic modification step (Figure S1, Supporting Information File 1) [25].

### Identification of new fabclavine derivatives

To analyze the identified *fcl* BGCs, mutant strains were generated with a chemically inducible promoter in front of *fclC* or corresponding homologues (Figure 2). The inducible promoter was integrated via conjugation, with *Escherichia coli* as a donor strain, followed by homologous recombination as described previously [14,22]. This led to a formal ‘knock out’ of the BGC and no production of the respective natural product without induction, whereas induced mutants showed mostly an overproduction of the respective natural product [14]. Initially, the non-induced promoter-exchange mutant was compared with the induced mutant and the wild type to identify signals, related to possible biosynthesis products of the *fcl* BGCs using the known structure of **1** as a reference [20]. To confirm these signals as fabclavine derivatives, high-resolution MALDI–MS measurements to determine the exact mass and MALDI–MS^2^ fragmentation patterns of selected derivatives were acquired. If necessary, the measurements were repeated from mutants cultivated in ^13^C media in order to determine the number of carbons in the sum formula [28].

The general structure of the fabclavines is highly conserved and differs only in the specified moieties as shown in Table 1. The NRPS part of the full-length fabclavines harbors six amino acids, whereby the second position (R^1^) varies between phenylalanine (Phe), histidine (His), and alanine (Ala) and the sixth position (R^2^/R^3^) between proline (Pro), valine (Val) and threonine (Thr). The polyamine can differ in the length from three to five amine units (*m*) and is connected via one to three partially reduced polyketide C2 units (*n*) with the NRPS part.

**Table 1:** Compound list of the fabclavine derivatives identified in this work. The structures are based on MALDI–HRMS and MALDI–MS^2^ analyses using the known structure of **1** as a reference [20]. The derivatives **1**–**4** and **17**–**22** were described previously [20].

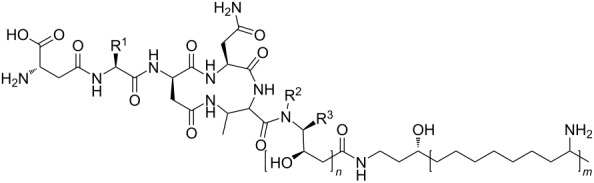							

#	R^1^	R^2^	R^3^	*n*	*m*	molecular formula	*m*/*z* [M + H]^+^

**1**	Bn	–(CH_2_)_3_–		2	4	C_70_H_125_N_13_O_13_	1356.9593
**2**	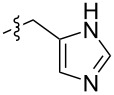	–(CH_2_)_3_–		2	4	C_67_H_123_N_15_O_13_	1346.9498
**3**	Bn	–(CH_2_)_3_–		1	4	C_68_H_121_N_13_O_12_	1312.9330
**4**	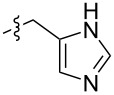	–(CH_2_)_3_–		1	4	C_65_H_119_N_15_O_12_	1302.9235
**5**	Bn	–(CH_2_)_3_–		2	3	C_62_H_108_N_12_O_13_	1229.8232
**6**	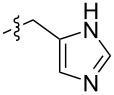	–(CH_2_)_3_–		2	3	C_59_H_106_N_14_O_13_	1219.8137
**7**	Bn	–(CH_2_)_3_–		1	3	C_60_H_104_N_12_O_12_	1185.7969
**8**	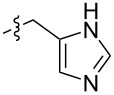	–(CH_2_)_3_–		1	3	C_57_H_102_N_14_O_12_	1175.7874
**9**	Bn	–(CH_2_)_3_–		2	5	C_78_H_142_N_14_O_13_	1484.0954
**10**	Bn	–(CH_2_)_3_–		1	5	C_76_H_138_N_14_O_12_	1440.0691
**11**	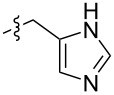	–(CH_2_)_3_–		2	5	C_75_H_140_N_16_O_13_	1474.0859
**12**	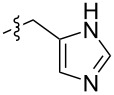	–(CH_2_)_3_–		1	5	C_73_H_136_N_16_O_12_	1430.0596
**13**	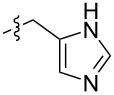	H	iPr	1	5	C_73_H_138_N_16_O_12_	1432.0753
**14**	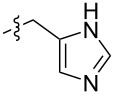	H	iPr	2	5	C_75_H_142_N_16_O_13_	1476.1015
**15**	CH_3_	H	iPr	1	3	C_54_H_102_N_12_O_12_	1111.7813
**16**	CH_3_	H	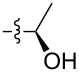	1	3	C_53_H_100_N_12_O_13_	1113.7606
**17**	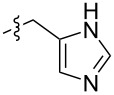	H	iPr	1	3	C_57_H_104_N_14_O_12_	1177.8031
**18**	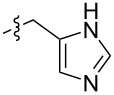	H	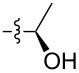	1	3	C_56_H_102_N_14_O_13_	1179.7824
**19**	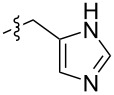	H	iPr	2	3	C_59_H_108_N_14_O_13_	1221.8293
**20**	Bn	H	iPr	1	3	C_60_H_106_N_12_O_12_	1187.8126
**21**	Bn	H	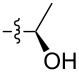	1	3	C_59_H_104_N_12_O_13_	1189.7979
**22**	Bn	H	iPr	2	3	C_62_H_110_N_12_O_13_	1231.8388
**23**	Bn	H	iPr	1	4	C_68_H_123_N_13_O_12_	1314.9487
**24**	Bn	H	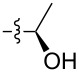	1	4	C_67_H_121_N_13_O_13_	1316.9280
**25**	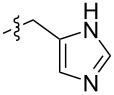	H	iPr	1	4	C_65_H_121_N_15_O_12_	1304.9392
**26**	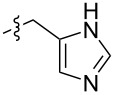	H	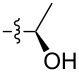	1	4	C_64_H_119_N_15_O_13_	1306.9185
**27**	Bn	H	iPr	2	4	C_70_H_127_N_13_O_13_	1358.9749
**28**	Bn	H	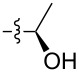	2	4	C_69_H_125_N_13_O_14_	1360.9542
**29**	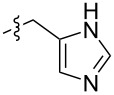	H	iPr	2	4	C_67_H_125_N_15_O_13_	1348.9654
**30**	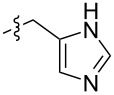	H	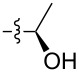	2	4	C_66_H_123_N_15_O_14_	1350.9447
**31**	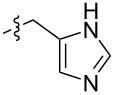	H	iPr	3	4	C_69_H_129_N_15_O_14_	1392.9916
**32**	Bn	H	iPr	3	4	C_72_H_131_N_13_O_14_	1403.0011

In this work, 22 yet unknown derivatives could be identified, which led to a total of 32 full-length derivatives (Table 1). In the following, the fabclavine characteristics of the individual or multiple strains are highlighted.

Besides derivatives with a polyamine of four amine units (**1**–**4**), the already described fabclavine producer *X. budapestensis* showed also the incorporation of a three-amine unit polyamine (**5**–**8**, Table 1 and Figure S7, Supporting Information File 1). A similar set of derivatives could be observed for the closely related strains *X. indica* and *X. cabanillasii* (Table 1 and Figures S11, S12, S15, and S16, Supporting Information File 1). In these strains, additional derivatives with polyamines made of five amine units were identified (**9**–**12**, Table 1 and Figures S13 and S17, Supporting Information File 1).

In *X. hominickii*, only derivatives with polyamines made of five amine units were identified, but none with less (Table 1). Here, the polyamine was connected by one or two polyketide units to a NRPS part, with His in the second (Table 1, R^1^) and Pro or Val in the sixth amino acid position (Table 1, R^2^/R^3^ and Figure S18, Supporting Information File 1), leading to the smallest set of identified derivatives (**11**–**14**).

In *X. szentirmaii*, derivatives with an Ala incorporated at the second amino acid position were identified (**15** and **16**, Table 1, R^1^) in addition to the already described derivatives **17**–**22**. However, the derivatives featuring the described Ala incorporation were not detected in any other strain analyzed (Table 1 and Figure S2, Supporting Information File 1) [20]. Furthermore, besides the dominant derivatives with a Val or Thr residue in the sixth amino acid position (Table 1, R^2^/R^3^), derivatives containing Pro were also observed, but with a much lower signal intensity (Figure S3, Supporting Information File 1).

As expected, the close taxonomic relationship between *X. stockiae*, KJ12.1 and KK7.4 resulted also in a similar set of produced fabclavine derivatives: Here, a *X. szentirmaii*-similar NRPS-derived part was connected to a polyamine with four amine units (**23**–**30**, Table 1). A special feature of this group were derivatives with up to three incorporated polyketide units instead of the usual one or two (**31** and **32**, Table 1 and Figure S25, Supporting Information File 1). Further signals were detected with a low abundance, suggesting the incorporation of Pro as a sixth amino acid (Figures S21, S26, and S29, Supporting Information File 1).

For *P. temperata* and *X. bovienii*, no fabclavine derivatives could be detected (data not shown), probably resulting from the missing NRPS and PKS genes (Figure 2).

After the identification of the full-length derivatives, all strains were analyzed for the presence of shortened fabclavines, previously identified in *X. szentirmaii* [22]. Therefore, their structure was predicted from the elucidated full-length derivatives (Table 2). Surprisingly, only in *X. szentirmaii*, the abundance of compounds with 715 and 717 Da could be clearly confirmed (Figure S4, Supporting Information File 1). In the other strains, the expected shortened derivatives were not detectable (data not shown).

**Table 2:** Occurrence of the different fabclavine derivatives in the analyzed *Xenorhabdus* strains. The results are based on the MALDI–HRMS and MALDI–MS^2^ analyses shown in Figures S2–S29 (Supporting Information File 1).

strain	compound

*X. budapestensis*	**1**–**8**
*X. indica*	**1**–**12**
*X. cabanillasii*	**1**–**4**, **8**, **11**, **12**
*X. hominickii*	**11**–**14**
*X. szentirmaii*	**15**–**22**
KJ12.1	**23**–**32**
*X. stockiae*	**23**–**32**
KK7.4	**2**, **23**–**32**
*X. innexi*	**4**, **23**–**32**

Our observation indicates that all strains produce additional derivatives than described in Table 2. Due to the fact that some of these derivatives were hardly detectable, preventing a structure confirmation and elucidation by MALDI–MS^2^, they are only shown as proposed minor derivatives in the supplementary results (Figure S33, Supporting Information File 1).

As we were not able to generate a promoter-exchange mutant in *X. innexi* DSM 16336, its fabclavine derivatives were identified in the wild type. MALDI–HRMS measurements revealed multiple signals corresponding to fabclavines (Figures S30 and S31, Supporting Information File 1). To confirm the signal at 1392.99 Da as corresponding to compound **31**, a MALDI–MS^2^ analysis was performed, resulting in the characteristic fragment ions with 598 Da for the polyamine part, and 795 Da for the NRPS-PKS part (Figure S32, Supporting Information File 1). Considering the fragmentation pattern for compound **31** and standard deviations below 1.3 ppm for further fabclavine derivatives, *X. innexi* could indeed be confirmed as a producer of fabclavines similar to those from *X. stockiae* (Figures S30–S32, Supporting Information File 1).

### Bioactivity of the different fabclavine producers

Previous studies revealed that the fabclavines show a broad-spectrum bioactivity against a variety of different organisms [20]. To verify the bioactivity of the derivatives described in this work, the inhibitory activity of the wild type and the promoter-exchange mutants (induced and non-induced) were analyzed against the human pathogens *Escherichia coli*, *Staphylococcus aureus*, *Enterococcus faecalis*, and *Klebsiella pneumoniae* by agar well-diffusion bioassays (Table 3). Briefly, cell-free supernatant was filled into wells of agar plates, which were inoculated with the pathogenic bacteria. Subsequently, the diameters of the inhibition zones were measured after 48 h. As references, different kanamycin concentrations to generate comparable inhibition zones were used (Table S4, Supporting Information File 1).

**Table 3:** Inhibition zones of the wild type (WT) and promoter-exchange mutant strains (non-ind: non-induced, ind: induced) in mm against the human pathogens *Escherichia coli* (a, ATCC 25922), *Staphylococcus aureus* (b, ATCC 29213), *Enterococcus faecalis* (c, ATCC 29212), and *Klebsiella pneumoniae* (d, ATCC 700603). The corresponding agar well-diffusion bioassays were performed three times, with ten replicates for each sample. *X. sto*. = *X. stockiae*, *X. ind*. = *X. indica*, *X. hom*. = *X. hominickii*, *X. sze*. = *X. szentirmaii*, *X. cab*. = *X. cabanillasii*, *X. bud*. = *X. budapestensis*.

	sample	strain							
		KJ12.1	KK7.4	*X. sto.*	*X. ind.*	*X. hom.*	*X. sze.*	*X. cab.*	*X. bud.*

a	WT	12.4 ± 0.2	12.6 ± 0.1	11.4 ± 0.2	17.8 ± 0.2	10.2 ± 0.2	12.6 ± 0.2	18.4 ± 0.2	18.8 ± 0.2
	non-ind	0	0	0	9 ± 0.2	0	0	8.8 ± 0.2	0
	ind	12.4 ± 0.3	14.6 ± 0.3	16 ± 0.2	18.2 ± 0.2	11.8 ± 0.2	16.8 ± 0.3	18.8 ± 0.2	18.8 ± 0.2

b	WT	16.8 ± 0.2	17.2 ± 0.2	15.4 ± 0.2	23.4 ± 0.3	13.8 ± 0.2	14.8 ± 0.2	22.6 ± 0.2	21.8 ± 0.2
	non-ind	0	0	0	0	0	0	0	0
	ind	16.6 ± 0.2	19.6 ± 0.2	21 ± 0.3	25 ± 0.2	16 ± 0.2	20.8 ± 0.2	22.6 ± 0.2	22.4 ± 0.2

c	WT	11.2 ± 0.3	14.4 ± 0.2	12.6 ± 0.2	17 ± 0.2	13.2 ± 0.2	11.5 ± 0.3	19.4 ± 0.3	16.6 ± 0.3
	non-ind	0	0	0	0	0	0	0	0
	ind	14.6 ± 0.2	17.2 ± 0.3	18.6 ± 0.2	19.6 ± 0.3	15.6 ± 0.4	11.8 ± 0.2	20.4 ± 0.3	18.2 ± 0.2

d	WT	13.5 ± 0.2	13 ± 0.2	8.4 ± 0.3	18 ± 0.1	9.2 ± 0.2	10.7 ± 0.2	18.1 ± 0.2	13.3 ± 0.2
	non-ind	0	0	0	0	0	0	0	0
	ind	14.3 ± 0.2	15.2 ± 0.2	16.4 ± 0.1	20.3 ± 0.2	12.6 ± 0.2	12 ± 0.2	22.2 ± 0.3	18.4 ± 0.2

All analyzed wild type strains showed inhibition zones against the selected pathogens. Additionally, a comparison of the induced and the non-induced promoter-exchange mutants confirmed that the main bioactivity of all strains strongly depends on the fabclavines (Table 3). Interestingly, the non-induced promoter-exchange mutants of *X. cabanillasii* and *X. indica* showed an additional bioactivity, which might be due to another bioactive compound class (Table 3).

## Discussion

Together with the ten previously published fabclavine derivatives, in total 32 fabclavines were identified in this work, which can be extended to 37 if the minor derivatives are included as well (Table 1 and Figure S33, Supporting Information File 1). As variable positions in the general structure, the second (Phe, His, Ala) and sixth amino acid position (Pro, Val, Thr) were identified as well as one to three partially reduced polyketide units or three to five amine units in the polyamine part. Combining all four variable positions in the general structure, 81 different fabclavine derivatives are theoretically possible. Strikingly, except for some minor derivatives, each strain or group of strains has its own set of fabclavines with unique features, such as polyamines with different lengths or an additional polyketide unit.

Considering the fabclavine biosynthesis in *X. szentirmaii*, the responsible components for such a chemical variety seem to be the following: The first is a lowered substrate specificity of two A-domains A_2_ and A_6_ in the NRPSs FclI and FclJ (Figure 1) [22]. Surprisingly, the key residues of these domains are highly conserved or identical, even between strains that differ in the incorporated amino acids (Table S6, Supporting Information File 1). This indicates the involvement of further structural elements, such as C-domains for the amino acid specificity [29–30]. However, an A-domain promiscuity is common in NRPS, exemplified by the biosynthesis of microcystins from cyanobacteria, RXPs or xenematide from *Xenorhabdus* and *Photorhabdus* [31–33]. The second strategy includes the iterative use of the PKSs FclK, responsible for the elongation with polyketide units, and FclC, responsible for the generation of the polyamine (Figure 1) [22]. As described previously, the genes *fclC*, *fclD*, and *fclE* are related to the PUFA biosynthesis genes and are responsible for the polyamine formation [20]. As this biosynthesis is based on iterative cycles, the polyamine biosynthesis shows a similar pattern [22,34–35]. The elongation with one to three malonate units by the type I PKS FclK for product diversification is unusual. However, multiple examples for bacterial iterative type I PKS are known, such as enediynes, myxochromide, aureothin, micacocidin, and further SMs [36–42].

Multiple fabclavine derivatives were identified in *X. innexi* DSM 16336 by MALDI–MS experiments in combination with the generated compound list. According to the literature, the *X. innexi* strains HGB1681 and HGB1997 are responsible for the biosynthesis of Xlt with a major range of 1348 to 1402 Da, similar to that of the fabclavines identified in this work [27]. Furthermore, the strains KJ12.1, KK7.4, *X. stockiae*, and *X. innexi* can be phylogenetically grouped together, and our results show that taxonomically related strains also produce similar sets of fabclavines (Table 2) [26]. In addition to the high homology between the *xlt* and *fcl* BGCs, our results strongly suggest that Xlt and the fabclavines are identical. The bioactivity described for Xlt relies on the induction of epithelial cell apoptosis in the anterior midgut of larvae [43]. Consequently, this mode of action could also be possible for fabclavines.

## Conclusion

This study revealed a large chemical diversity for fabclavine derivatives among different *Xenorhabdus* strains, which is achieved by the promiscuity of single enzymes or domains during the biosynthesis. The recently published “easy promoter-activated compound identification” approach utilizes mutants with a deletion of the chaperone Hfq, leading to a loss of SM production [15]. Subsequent reactivation of selected BGCs results in an almost exclusive production of one compound class, and the corresponding study revealed that fabclavines alone are the major bioactive compound class in *X. szentirmaii* [15]. In combination with our bioactivity data of fabclavine-producing mutants, it is obvious that this class of compounds is the major driver for the overall antibiotic activity against the tested Gram-positive and Gram-negative bacteria in the other strains analyzed (Table 3). Whether this bioactivity is due to individual members of the fabclavines or whether all of them have a comparable activity must be studied in the future after the isolation of the individual derivatives.

Nevertheless, synergistic effects with other compound classes, enhancing the overall inhibitory activity, cannot be excluded. As an example, *X. indica* and *X. cabanillasii* showed an additional bioactivity against Gram-negative bacteria even without fabclavine production (Table 3). This bioactivity might be caused by other compound(s) as both strains have the potential to produce further bioactive SMs, such as cabanillasin, PAX peptides, or rhabdopeptides [26,44–46], which will be studied in the future. Furthermore, the identification of fabclavine derivatives described here might support recent studies that revealed *Xenorhabdus* and *Photorhabdus* strains having ascaricidal or larvicidal activity. Here, especially *X. szentirmaii*-, *X. indica*-, *X. stockiae*-, as well as *X. stockiae*-related isolates showed the best activity [18–19,47]. Although these strains were confirmed as fabclavine producers in our current study, future work is required to confirm fabclavines as the active compounds here as well (Table 3) [20].

## Supporting Information

File 1 Material and methods, supplementary figures and tables, and MALDI–HRMS and MALDI–MS^2^ spectra.
