# Supplementary material for: Fabclavine diversity in Xenorhabdus bacteria
Source: Beilstein J Org Chem. 2020 May 7;16:956–65. doi: 10.3762/bjoc.16.84 (PMC7214866; doi:10.3762/bjoc.16.84)
Supplement: File 1 — Material and methods, supplementary figures and tables, and MALDI–HRMS and MALDI–MS2 spectra. [file Beilstein_J_Org_Chem-16-956-s001.pdf]

BEILSTEIN JOURNAL OF ORGANIC CHEMISTRY

Supporting Information

for

Fabclavine diversity in *Xenorhabdus* bacteria

Sebastian L. Wenski, Harun Cimen, Natalie Berghaus, Sebastian W. Fuchs, Selcuk Hazir and Helge B. Bode

Beilstein J. Org. Chem. **2020,** *16,* 956–965. doi:10.3762/bjoc.16.84

Material and methods, supplementary figures and tables, and MALDI–HRMS and MALDI–MS^2 spectra

License and Terms: This is a supporting information file under the terms of the Creative Commons Attribution License (http://creativecommons.org/licenses/by/4.0). Please note that the reuse, redistribution and reproduction in particular requires that the authors and source are credited.
The license is subject to the *Beilstein Journal of Organic Chemistry* terms and conditions: (https://www.beilstein-journals.org/bjoc)

Material and methods

1. Strain cultivation for MALDI–MS experiments

The strains were cultivated as described previously [1]: Briefly, all *Xenorhabdus* and *Photorhabdus* strains were grown on lysogeny broth (LB) agar plates at 30 °C. The production cultures were inoculated 1:25 with overnight cultures in fresh LB medium and incubated shaking at 30 °C for three days. *Escherichia coli* strains were grown on LB agar plates and in LB medium at 37 °C with shaking. If appropriate, kanamycin (50 µg/mL) or L-(+)-arabinose (0.2%) were added. The cultures were harvested as whole cell cultures and stored at −20 °C.

2. Isotope labeling and reverse feeding experiments

For the isotope-labeling experiments, overnight cultures were washed three times with H_2O and inoculated 1:100 in ISOGRO®-13C (Sigma-Aldrich). If appropriate, kanamycin and L-(+)-arabinose (0.2%) were added, and the cultures were incubated shaking at 30 °C for 2 d. For reverse feeding experiments, 2 mM of the substrate was added each day.

3. Generation of promoter-exchange mutants

The promoter-exchange mutants were generated as described previously [1]: Briefly, about 600–1200 bp of the starting *fclC* (homologues) were amplified (the corresponding oligonucleotides are listed in Table S1), cloned into the pCEP_Kan backbone (pCEP: cluster expression plasmid) by hot fusion assembly, and transformed into *E. coli* S17-1 λpir or ST18 as described previously [2-5]. After the conjugation with the corresponding *E. coli* ST18/S17 strains, the kanamycin-resistant conjugants were confirmed by colony PCR or by MALDI–MS for a successful promoter exchange.

4. MALDI–MS

MALDI–MS experiments were performed as described previously [1]: Briefly, 0.3 µL of a liquid culture were mixed on a MALDI target with 0.25 µL 1:10 diluted ProteoMass Normal Mass Calibration Mix (ProteoMass™ MALDI Calibration Kit, Sigma-Aldrich) for internal calibration and 0.9 µL α-cyano-4-hydroxycinnamic acid (CHCA) matrix (3 mg/mL in 75% acetonitrile, 0.1% trifluoroacetic acid). After drying, the resulting spot was coated with 5% formic acid, and after removal of the 5% formic acid solution mixed again with 0.6 µL

CHCA. Cell MALDI measurements were performed with a MALDI LTQ Orbitrap XL (Thermo Fisher Scientific, Inc., Waltham, MA) instrument (nitrogen laser at 337 nm in FTMS scan mode) with 100 shots per measurement with high resolution. Alternatively, 4-chloro-α-cyanocinnamic acid can be used as the matrix, but we observed a decreased quality of the resulting spectra. The CID mode using the ITMS scan mode was used for MALDI–MS^2 experiments, with the same sample preparation having the following parameters: Normalized collision energy: 28–32, Act. Q: 0.250, Act. Time (ms): 30.0. The data were analyzed using Qual Browser version 2.0.7 (Thermo Fisher Scientific). As the signal intensities of the stacked spectra were normalized and the intensity is displayed by the relative abundance in a range from 0 to 100%, the y axis is not shown. Since not all detected signals could be detected with a sufficient signal intensity to confirm them by MS^2 experiments, we decided to show them in the supplementary figures of the corresponding strain, but without a compound number.

5. Bioactivity analysis

5.1 Generating cell-free supernatant

The promoter-exchange mutant strains were cultivated on LB agar, supplemented with a 50 µg/mL final concentration of kanamycin, and incubated at 30 °C for 48 h. A single colony was transferred into 10 mL LB medium, supplemented with a 50 µg/mL final concentration of kanamycin to obtain a culture overnight at 200 rpm and 30 °C. The optical densities of the overnight cultures (20 mL LB) were measured at 600 nm. The final OD of the cultures was adjusted to 0.1 after inoculation. For each strain, two flasks were prepared, and the cultures were incubated at 30 °C for 1 h. One of the flasks was induced with 0.2% L-arabinose, and the other flask was not treated with arabinose (non-induced). All induced and non-induced cultures were incubated for 24 h at 200 rpm and 30 °C. Wild type strains were grown on LB agar at 30 °C for 48 h. A single colony was transferred in 10 mL LB medium and incubated overnight at 30 °C in a rotary shaker at 200 rpm to generate precultures. The LB medium was inoculated 1:100 with the preculture and cultivated at 200 rpm and 30 °C for 72 h. The bacterial broth was harvested by centrifugation at 10,000 rpm for 10 min, and the supernatant was filtered through a 0.22 µm millipore filter (Thermo scientific).

5.2 Agar well-diffusion bioassay

The antibacterial activity of the wild type, induced, and noninduced promoter-exchange mutant supernatants was determined by the agar well-diffusion method [6]. *Escherichia coli* (ATCC 25922), *Enterococcus faecalis* (ATCC 29212), *Staphyllococcus aureus* (ATCC 29213), and *Klebsiella pneumoniae* (ATCC 700603) were used in the experiments. For the preparation of overnight cultures of the test organisms, 20 mL LB medium was inoculated with a loopful of the bacterium and incubated at 37 °C in a rotary shaker at 200 rpm. Briefly, 200 µL of the pathogen bacteria from an overnight culture was spread on a Mueller–Hinton agar (blood agar was used for *E. faecalis*) with a glass rod. Subsequently, three wells were made in each of these plates using a 5 mm diameter sterile transfer tube. Each well was filled with 60 µL of induced, non-induced mutant, or wild type of the bacterial supernatants. Kanamycin was used as a positive control at different concentrations (25, 50, 100, 200, 400, and 800 µL/mg). Petri dishes (10 mm) were incubated at 37 °C for 48 h. After the incubation period, the diameter of the inhibition zones (mm) was assessed by measuring the diameter in two perpendicular directions and taking the average [7]. Ten petri dishes were used for each replicate, and the experiments were conducted three times on different dates.

Supplementary material:

Table S1: Overview of oligonucleotides used in this work for the amplification of the homologous regions of the promoter-exchange mutants.

strain	locus tag(s) of analyzed gene(s)	oligonucleotide	sequence 5‘–3‘
***X. szentirmaii* pCEP_fcl**	Xsze_03745	SW159_XszC_fw	TTTGGGCTAACAGGAGGCTAGCATATGTCTGAGACATATTTTTTACATGATAGAAAAATTCGTGG
		SW160_XszC_rv	TCTGCAGAGCTCGAGCATGCACATGCAATATTCCCGCACGGGTATATGC
***X. budapestensis* pCEP_fcl**	Xbud_02634	SW271_Xbud_fw	TTTGGGCTAACAGGAGGCTAGCATATGTCCAAGACGTATTTTTTGCATG
		SW272_Xbud_rv	TCTGCAGAGCTCGAGCATGCACATCTTCATTCCACTCAAGATACATTCCG
***X. indica* pCEP_fcl**	Xind_02757	SW132_Xind_fw	TTTGGGCTAACAGGAGGCTAGCATATGTCCAAGACGTATTTTTTGCATG
		SW133_Xind_rv	TCTGCAGAGCTCGAGCATGCACATCGCTTTTACCTGCCCTTCC
***X. hominickii* pCEP_fcl**	Xhom_02793	SW130_Xhom_fw	TTTGGGCTAACAGGAGGCTAGCATATGTCTGAGTCATATCTTTTACATGATGG
		SW131_XHom_rv	TCTGCAGAGCTCGAGCATGCACATCTTCAACTAATTGAATATCGCTCGG
***X. stockiae* pCEP_fcl**	Xsto_00102	SW273_uni_fw	TTTGGGCTAACAGGAGGCTAGCATATGTCCAGGACATATTTTTTGCATG
		SW276_Xsto_rv	TCTGCAGAGCTCGAGCATGCACATGCCAAGATCAAAATAACTGGCG
***X.* KJ12.1 pCEP_fcl**	Xekj_00388	SW153_KJ12.1_fw	TTTGGGCTAACAGGAGGCTAGCATATGTCCAGGACATATTTTTTGCATG
		SW154_KJ12.1_rv	TCTGCAGAGCTCGAGCATGCACATCCATCACTGGAAGCTTCAACG
***X.* KK7.4 pCEP_fcl**	ctg22_41	SW155_KK7.4_fw	TTTGGGCTAACAGGAGGCTAGCATTTGCATGACAGAAAAATTAATGGAG
		SW156_KK7.4_rv	TCTGCAGAGCTCGAGCATGCACATGTGCAAAAATACTCTTTGCACGG
***P. temperata* pCEP_fcl**	MEG1DRAFT_01183	SW267_Ptemp_fw	TTTGGGCTAACAGGAGGCTAGCATATGTCTGAGACATATTTAATGCGTGG
		SW268_Ptemp_rv	TCTGCAGAGCTCGAGCATGCACATAAGTTGCTTTTCGACAGTGCC

Table S2: Strains used in this work.

strain	#	description	origin
X. szentirmaii	DSM 16338	Wild type	[8]
X. budapestensis	DSM 16342	Wild type	[8]
X. cabanillasii	JM26	Wild type	[9]
X. indica	DSM 17382	Wild type	[10]
X. hominickii	DSM 17903	Wild type	[9]
X. stockiae	DSM 17904	Wild type	[9]
X. KJ12.1	KJ12.1	Wild type	[11]
X. KK7.4	KK7.4	Wild type	[12]
X. innexi	DSM 16336	Wild type	[8]
P. temperata	meg1	Wild type	[13]
X. szentirmaii pCEP_fcl	DSM 16338	Promoter exchange in front of Xsze_03745	This work
X. budapestensis pCEP_fcl	DSM 16342	Promoter exchange in front of Xbud_02634	This work
X. cabanillasii pCEP_fcl	JM26	Promoter exchange in front of Xcab_02060	[14]
X. indica pCEP_fcl	DSM 17382	Promoter exchange in front of Xind_02757	This work
X. hominickii pCEP_fcl	DSM 17903	Promoter exchange in front of Xhom_02793	This work
X. stockiae pCEP_fcl	DSM 17904	Promoter exchange in front of Xsto_00102	This work
X. KJ12.1 pCEP_fcl	KJ12.1	Promoter exchange in front of Xekj_00388	This work
X. KK7.4 pCEP_fcl	KK7.4	Promoter exchange in front of ctg22_41	This work
P. temperata pCEP_fcl	meg1	Promoter exchange in front of MEG1DRAFT_01183	This work
E. coli	S17 λ1-pir	used for conjugation	[3]
E. coli	ST18	used for conjugation	[4]
E. coli	ATCC25922	Bioactivity analysis	ATCC
Enterococcus faecalis	ATCC29212	Bioactivity analysis	ATCC
Staphyllococcus aureus	ATCC29213	Bioactivity analysis	ATCC
Klebsiella pneumoniae	ATCC700603	Bioactivity analysis	ATCC

Table S3: Comparison of protein identities [%] from FclA to FclN in *X. budapestensis* DSM 16342 with *X. cabanillasii* JM26 (A) and *X. indica* DSM 17382 (B). Protein alignments were performed by ClustalW alignment with the CostMatrix BLOSUM in Geneious 6.1.8.

Identity	FclA	FclB	FclC	FclD	FclE	FclF	FclG	FclH	FclI	FclJ	FclK	FclL	FclM	FclN
A	96.5	92.2	95.6	94.8	97.2	96.1	96.1	97.4	93.6	96.4	93.6	92.8	98.7	97.8
B	97.1	91.6	95.4	95.6	96.3	97.3	94.9	97.4	93.5	94.7	93.8	94.1	97.8	97

Table S4: Diameters of inhibition zones of different kanamycin concentrations against *Escherichia coli* (ATCC 25922), *Enterococcus faecalis* (ATCC 29212), *Staphyllococcus aureus* (ATCC 29213) and *Klebsiella pneumoniae* (ATCC 700603).

	**kanamycin concentration [µg/ml]**					
	**25**	**50**	**100**	**200**	**400**	**800**
E. coli	13.5	16.9	20.1	23.0	25.3	28.2
S. aureus	0.0	0.0	13.3	18.5	21.6	24.7
E. faecalis	0.0	0.0	11.9	16.5	19.3	24.1
K. pneumoniae	0.0	0.0	0.0	0.0	11.7	15.3

Table S5: Overview of the NCBI accession numbers of the strains used in this study.

**strain**	**accession number**
***Xenorhabdus szentirmaii* DSM 16338**	NIBV00000000
***Xenorhabdus budapestensis* DSM 16342**	NIBS00000000
***Xenorhabdus cabanillasii* JM26**	NJGH00000000
***Xenorhabdus indica* DSM 17382**	NKHP00000000
***Xenorhabdus hominickii* DSM 17903**	NJAI00000000
***Xenorhabdus stockiae* DSM17904**	NJAJ00000000
***Xenorhabdus* KJ12.1**	NJCW00000000
***Xenorhabdus* KK7.4**	NJAH00000000
***Xenorhabdus bovienii* SS-2004**	FN667741
***Xenorhabdus innexi* DSM 16336**	NIBU00000000
***Photorhabdus temperata* subsp. *temperata* Meg1**	NZ_JGVH00000000

Table S6: Stachelhaus-codes and corresponding amino acid prediction of the promiscous A-domains A_2 and A_6 in the analyzed strains [15,16].

	**A-domain A_2**		**A-domain A_6**	
**strain**	**Stachelhaus**	**prediction**	**Stachelhaus**	**prediction**
X. budapestensis	DTWTLASVGK	phe	DAWFIGGTFK	val
X. indica	DTWTLASVGK	phe	DAWFIGGTFK	val
X. cabanillasii	DTWTLASVGK	phe	DAWFIGGTFK	val
X. hominickii	DTWTIASVGK	phe	DALFIGGTFK	val
X. szentirmaii	DVWTMSAVGK	ser	DALFIGGTFK	val
KJ12.1	DTWTIASVGK	phe	DAWFIGGTFK	val
X. stockiae	DTWTIASVGK	phe	DAWFIGGTFK	val
KK7.4	DTWTIASVGK	phe	DAWFVGGTFK	val
X. innexi	DTWTMASVGK	phe	DAWFVGGTFK	val

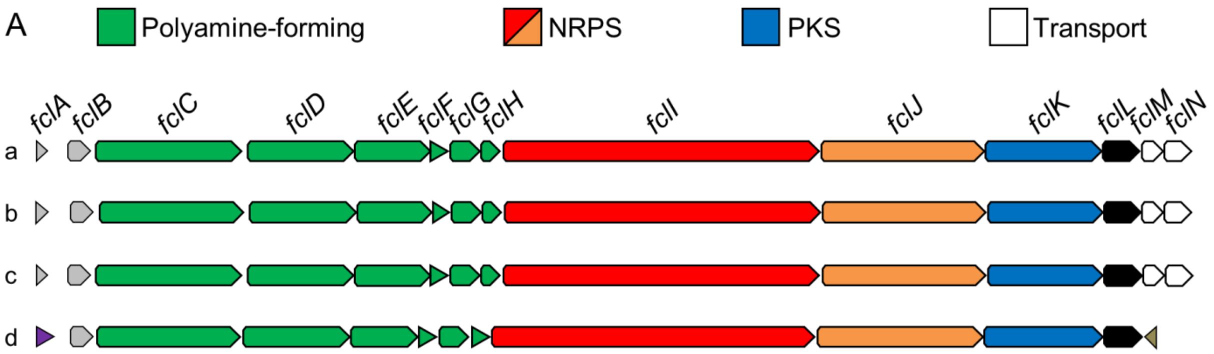

Identity	FclA	FclB	FclC	FclD	FclE	FclF	FclG	FclH	FclI	FclJ	FclK	FclL	FclM	FclN
b	98.8	95.5	96.3	96.1	96.3	100	99.5	98.9	95	97.4	95.4	97.3	98.7	98.4
c	100	99.4	99.2	98.4	99.6	100	100	99.2	98.2	99.3	99.3	99.6	99.7	99.7
d	-	68.7	75.9	77.5	82.1	90.6	81.2	85.7	72.9	78.6	73.9	74.6	-	-

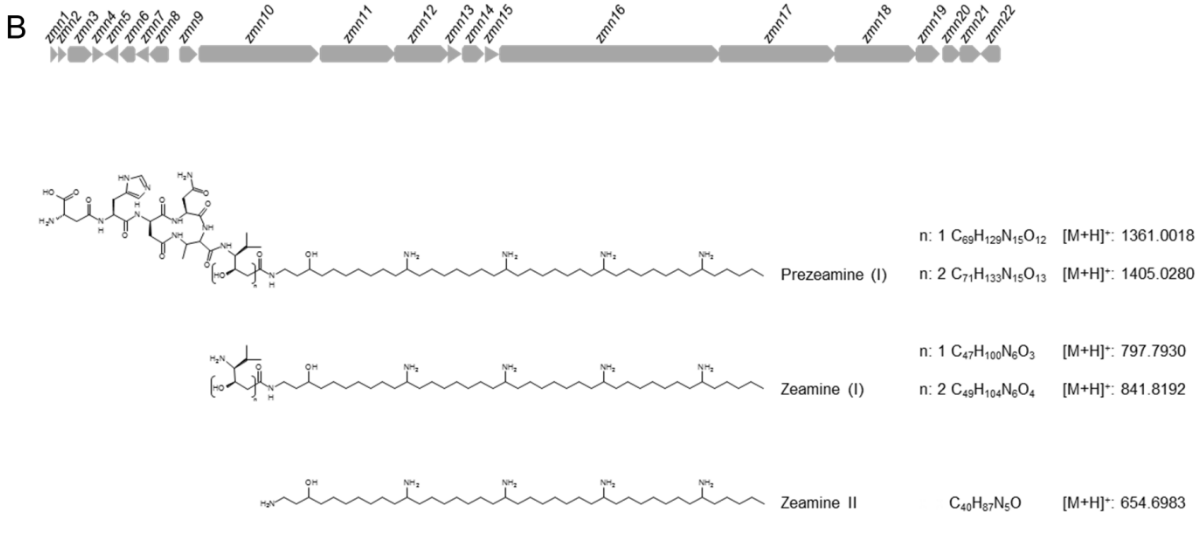

Figure S1: (A) Comparison of the *fcl* BGCs in *X. stockiae* DSM 17904 (a), KK7.4 (b), KJ12.1 (c) and the homologous gene cluster in *X. innexi* DSM 16336 (d). Shown are protein identities in comparison with X. stockiae [%]. Protein alignments were performed by ClustalW alignments with the CostMatrix BLOSUM in Geneious 6.1.8. The two aberrant genes of the *X. innxei* BGC are shown in purple (encoding a TonB homologue) and in brown (encoding an Acyl-CoA-thioesterase) [17]. (B) Zeamine biosynthesis gene cluster and corresponding compounds from *Serratia plymuthica* RVH1 [18,19].

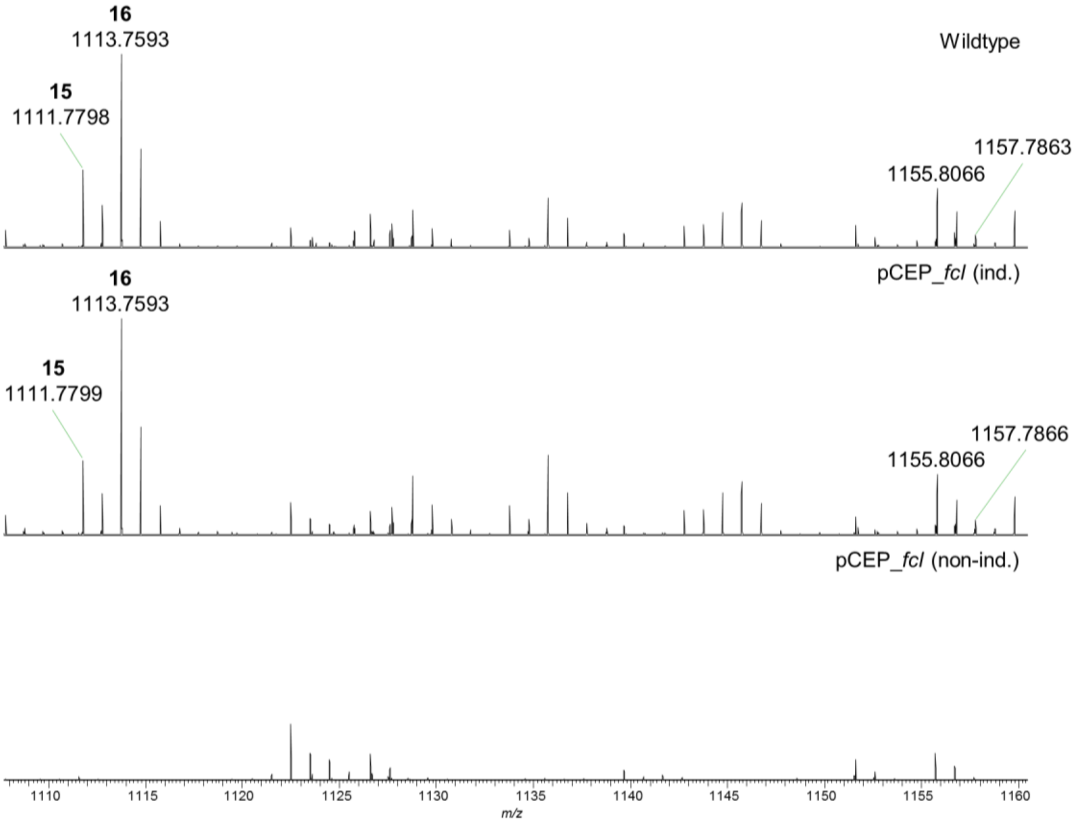

#	Sum formula	Calc. m/z	Det. m/z		Δppm	
			WT	pCEP_*fcl*	WT	pCEP_*fcl*
**15**	$C_{54}H_{102}N_{12}O_{12}$	1111.7813	1111.7798	1111.7799	-1.370	-1.244
**16**	$C_{53}H_{100}N_{12}O_{13}$	1113.7606	1113.7593	1113.7593	-1.120	-1.129
-	$C_{56}H_{106}N_{12}O_{13}$	1155.8075	1155.8066	1155.8066	-0.820	-0.759
-	$C_{55}H_{104}N_{12}O_{14}$	1157.7868	1157.7863	1157.7866	-0.408	-0.175

Figure S2: MALDI–HRMS spectra of *X. szentirmaii* wild type (WT) and pCEP_*fcl* promoter-exchange mutant (induced and noninduced) with compounds **15** and **16** showing sum formulas, calculated and detected masses and corresponding Δppm.

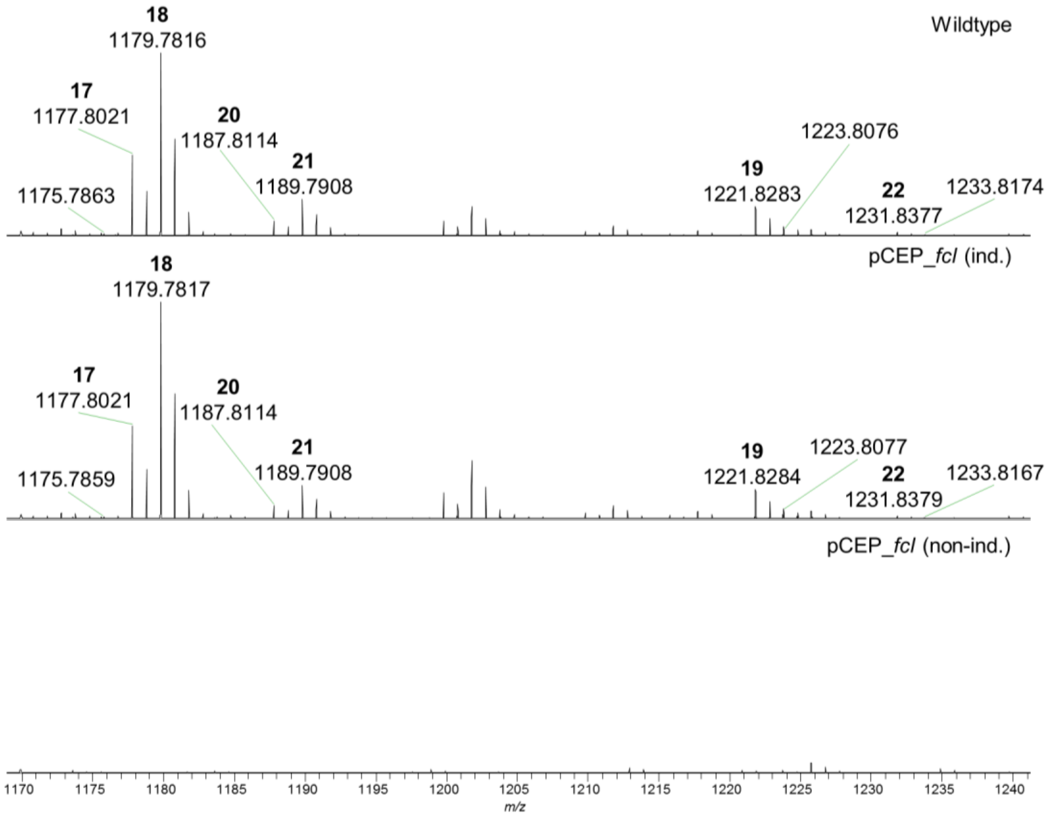

#	Sum formula	Calc. m/z	Det. m/z		Δppm	
			WT	pCEP_*fcl*	WT	pCEP_*fcl*
-	$C_{57}H_{102}N_{14}O_{12}$	1175.7874	1175.7863	1175.7859	-0.945	-1.294
**17**	$C_{57}H_{104}N_{14}O_{12}$	1177.8031	1177.8021	1177.8021	-0.850	-0.816
**18**	$C_{56}H_{102}N_{14}O_{13}$	1179.7824	1179.7816	1179.7817	-0.657	-0.539
**20**	$C_{60}H_{106}N_{12}O_{12}$	1187.8126	1187.8114	1187.8114	-1.013	-1.004
**21**	$C_{59}H_{104}N_{12}O_{13}$	1189.7979	1189.7908	1189.7908	-0.872	-0.914
**19**	$C_{59}H_{108}N_{14}O_{13}$	1221.8293	1221.8283	1221.8284	-0.839	-0.782
-	$C_{58}H_{106}N_{14}O_{14}$	1223.8086	1223.8076	1223.8077	-0.760	-0.736
**22**	$C_{62}H_{110}N_{12}O_{13}$	1231.8388	1231.8377	1231.8379	-0.875	-0.713
-	$C_{61}H_{108}N_{12}O_{14}$	1233.8181	1233.8174	1233.8167	-0.569	-1.104

Figure S3: MALDI–HRMS spectra of *X. szentirmaii* wild type (WT) and pCEP_*fcl* promoter-exchange mutant (induced and noninduced) with compounds **17–22** showing sum formulas, calculated and detected masses and corresponding Δppm. Compounds **17–22** were described previously [20].

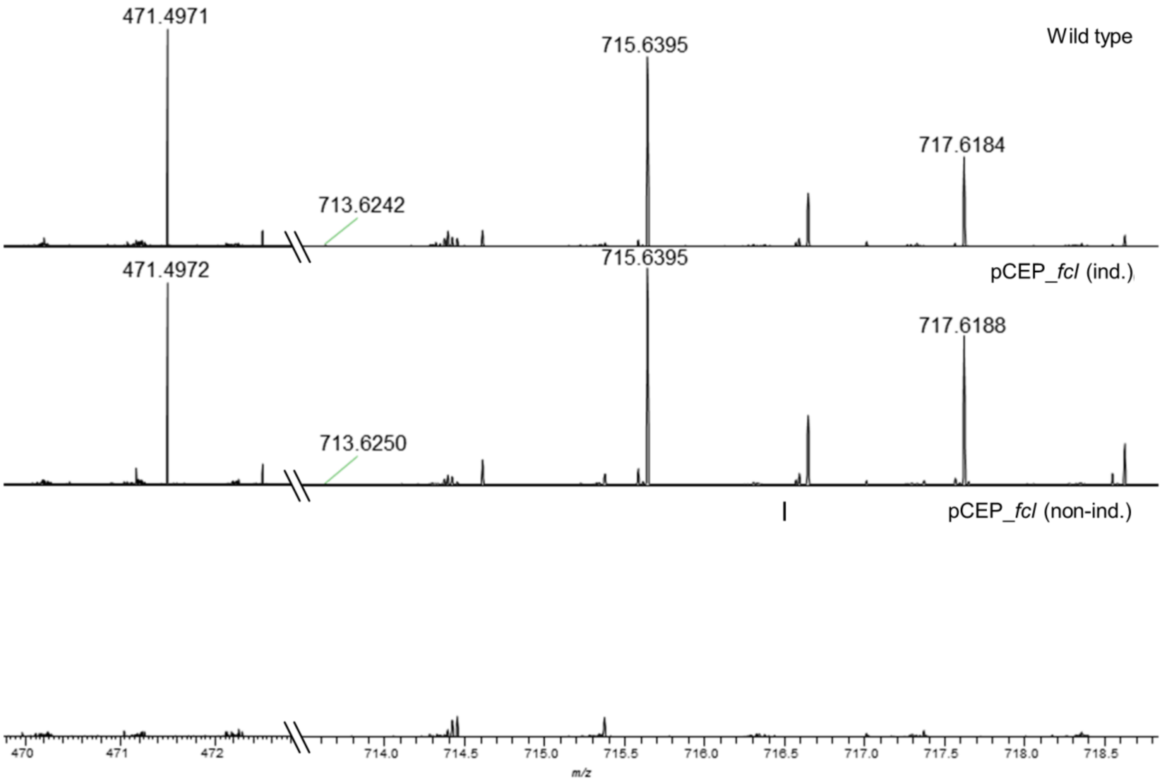

Description	Sum formula	Calc. m/z	Det. m/z		Δppm	
			WT	pCEP_*fcl*	WT	pCEP_*fcl*
Polyamine	$C_{28}H_{62}N_4O$	471.4996	471.4971	471.4972	-5.470	-5.237
Shortened Fabclavine	$C_{39}H_{80}N_6O_5$	713.6263	713.6243	713.6250	-2.867	-1.858
Shortened Fabclavine	$C_{39}H_{82}N_6O_5$	715.6419	715.6395	715.6395	-3.362	-3.474
Shortened Fabclavine	$C_{38}H_{80}N_6O_6$	717.6212	717.6184	717.6188	-3.861	-3.345

Figure S4: MALDI–HRMS spectra of *X. szentirmaii* wild type (WT) and pCEP_*fcl* promoter-exchange mutant (induced and noninduced) with polyamine and shortened fabclavines showing sum formulas, calculated and detected masses and corresponding Δppm.

Expected Fragmentions	Sum formula	m/z
y1	$C_{28}H_{63}N_4O$	471.4996
y2	$C_{35}H_{76}N_5O_3$	614.5943
y5	$C_{47}H_{93}N_{10}O_8$	925.7172
y6	$C_{50}H_{98}N_{11}O_9$	996.7544
b1	$C_4H_6NO_3$	116.0342
b2	$C_7H_{11}N_2O_4$	187.0713
b5	$C_{19}H_{28}N_7O_9$	498.1943
b6	$C_{26}H_{41}N_8O_{11}$	641.2889

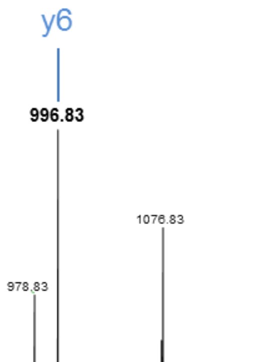

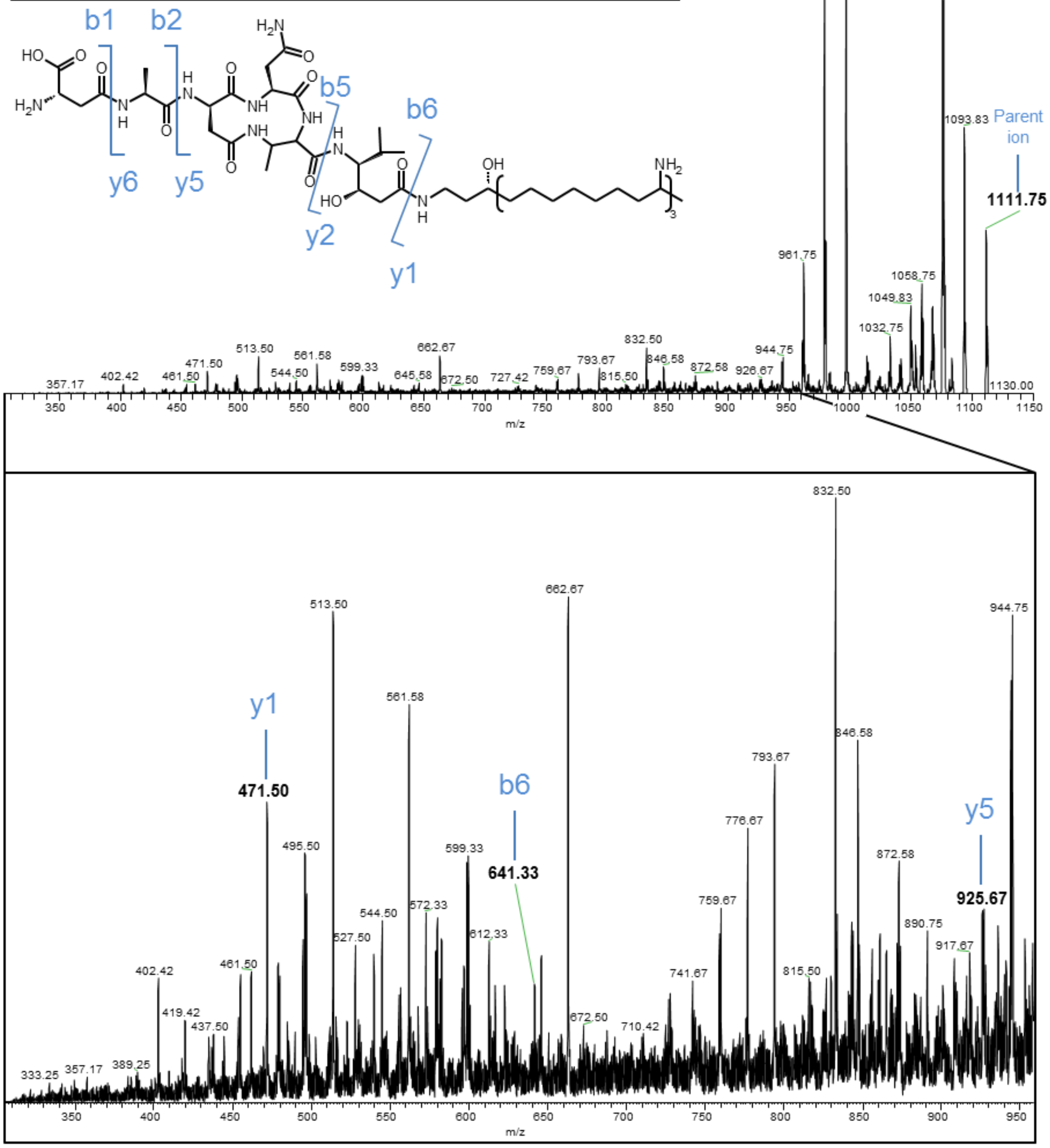

Figure S5: MALDI–MS^2 spectra of compound 1111.78 (**15**) of *X. szentirmaii* pCEP_*fcl* (induced) showing expected fragment ions and proposed structure.

Expected Fragmentions	Sum formula	m/z
y1	$C_{28}H_{63}N_4O$	471.4996
y2	$C_{34}H_{74}N_5O_4$	616.5735
y5	$C_{46}H_{91}N_{10}O_9$	927.6965
y6	$C_{49}H_{96}N_{11}O_{10}$	998.7336
b1	$C_4H_6NO_3$	116.0342
b2	$C_7H_{11}N_2O_4$	187.0713
b5	$C_{19}H_{28}N_7O_9$	498.1943
b6	$C_{25}H_{39}N_8O_{12}$	643.2682

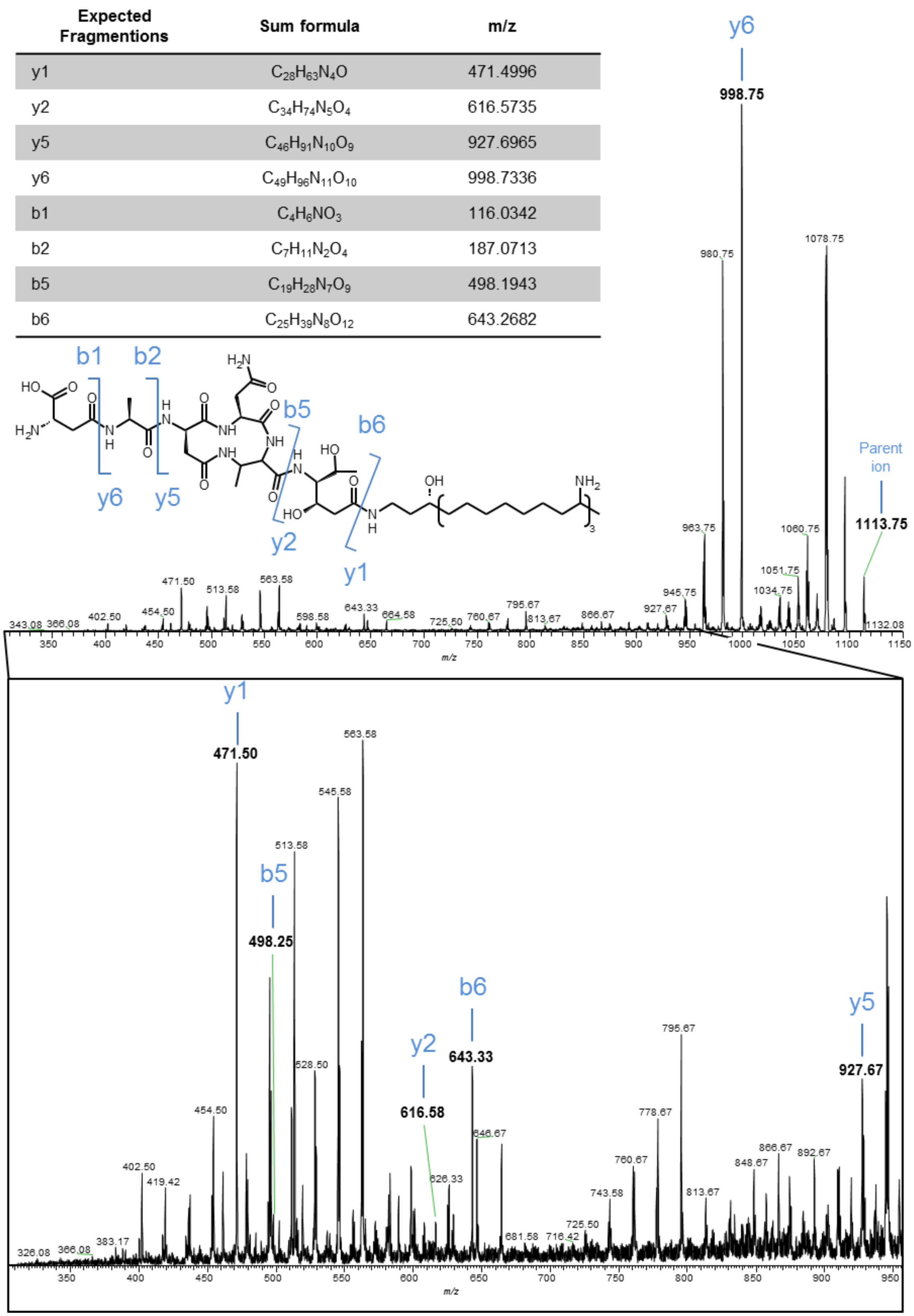

Figure S6: MALDI–MS^2 spectra of compound 1113.76 (**16**) of *X. szentirmaii* pCEP_*fcl* (induced) showing expected fragment ions and proposed structure.

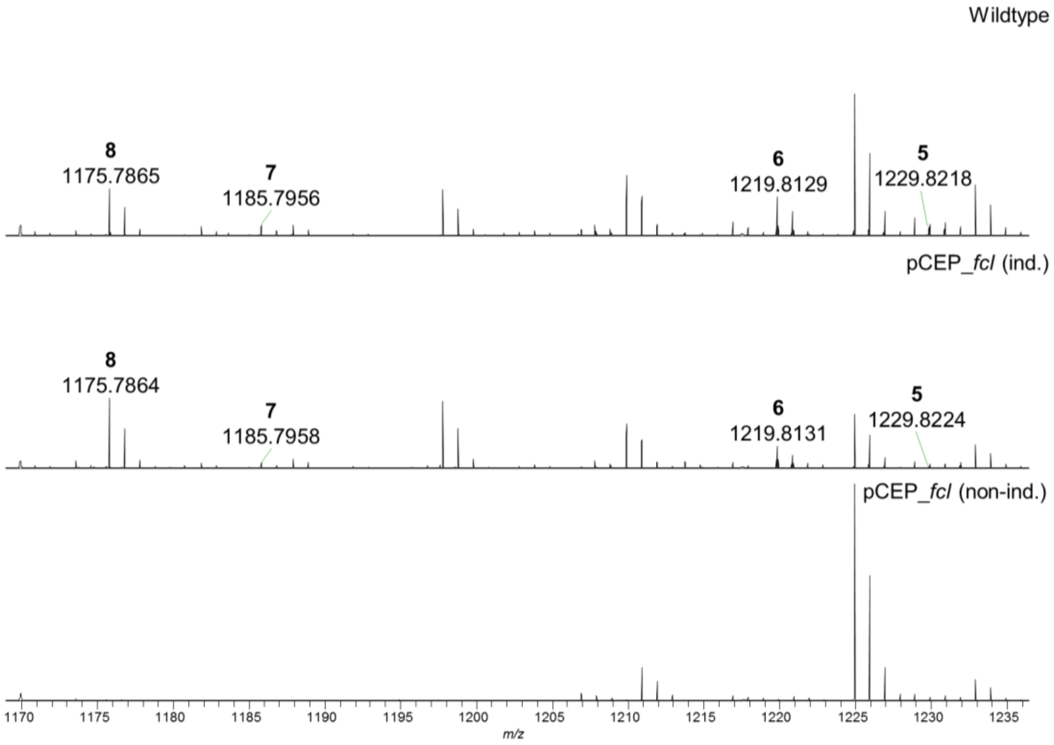

#	Sum formula	Calc. m/z	Det. m/z		Δppm	
			WT	pCEP_*fcl*	WT	pCEP_*fcl*
**8**	$C_{57}H_{102}N_{14}O_{12}$	1175.7874	1175.7865	1175.7865	-0.800	-0.843
**7**	$C_{60}H_{104}N_{12}O_{12}$	1185.7969	1185.7956	1185.7958	-1.107	-0.981
**6**	$C_{59}H_{106}N_{14}O_{13}$	1219.8137	1219.8129	1219.8131	-0.619	-0.480
**5**	$C_{62}H_{108}N_{12}O_{13}$	1229.8232	1229.8218	1229.8224	-1.088	-0.608

Figure S7: MALDI–HRMS spectra of *X. budapestensis* wild type (WT) and pCEP_*fcl* promoter-exchange mutant (induced and noninduced) with compounds **5**–**8** showing sum formulas, calculated and detected masses and corresponding Δppm.

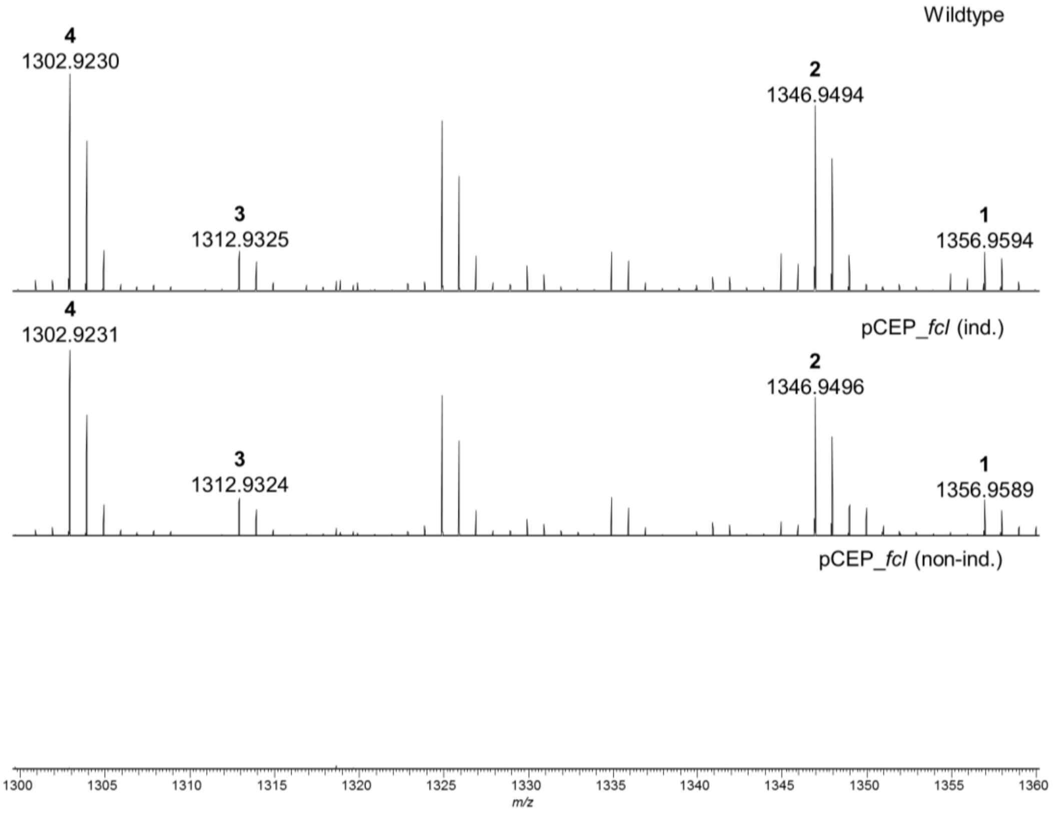

#	Sum formula	Calc. m/z	Det. m/z		Δppm	
			WT	pCEP_*fcl*	WT	pCEP_*fcl*
**4**	$C_{65}H_{119}N_{15}O_{12}$	1302.9235	1302.9230	1302.9231	-0.399	-0.338
**3**	$C_{68}H_{121}N_{13}O_{12}$	1312.9330	1312.9325	1312.9324	-0.451	-0.459
**2**	$C_{67}H_{123}N_{15}O_{13}$	1346.9498	1346.9494	1346.9496	-0.256	-0.100
**1**	$C_{70}H_{125}N_{13}O_{13}$	1356.9593	1356.9594	1356.9589	0.098	-0.248

Figure S8: MALDI–HRMS spectra of *X. budapestensis* wild type (WT) and pCEP_*fcl* promoter-exchange mutant (induced and noninduced) with compounds **1**–**4** showing sum formulas, calculated and detected masses and corresponding Δppm. Compounds **1**–**4** were described previously [20].

Expected Fragmentions	Sum formula	m/z
y1	$C_{28}H_{63}N_4O$	471.4996
y2	$C_{35}H_{74}N_5O_3$	612.5786
y5	$C_{47}H_{91}N_{10}O_8$	923.7016
y6	$C_{53}H_{98}N_{13}O_9$	1060.7605
b1	C_4H_6NO3	116.0342
b2	$C_{10}H_{13}N_4O_4$	253.0931
b5	$C_{22}H_{30}N_9O_9$	564.2161
b6	$C_{29}H_{41}N_{10}O_{11}$	705.2951

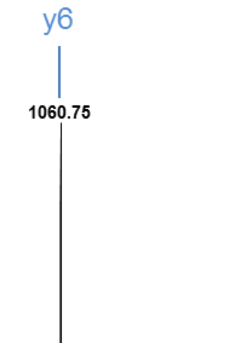

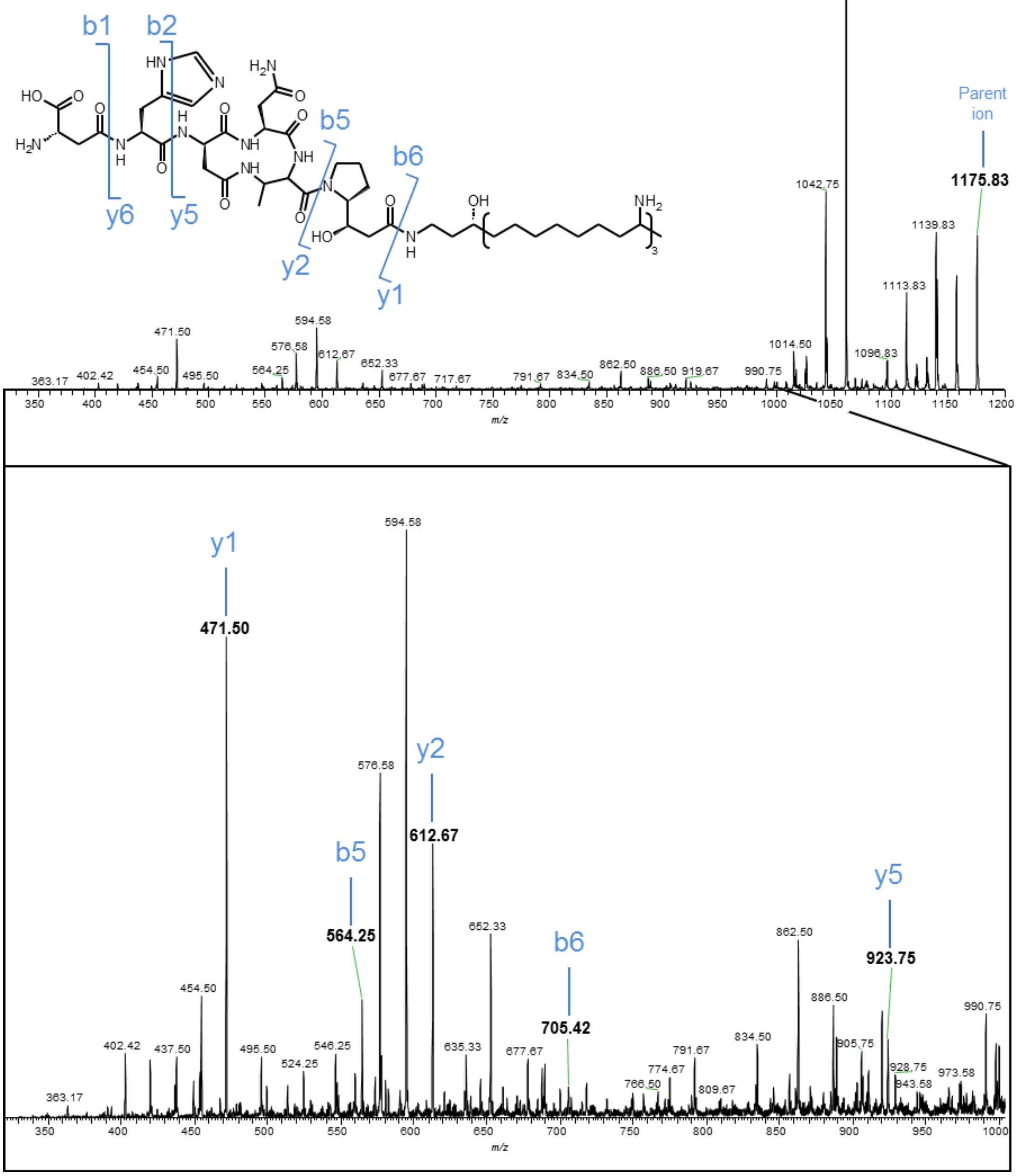

Figure S9: MALDI–MS^2 spectra of compound 1175.78 (**8**) of *X. budapestensis* pCEP_*fcl* (induced) showing expected fragment ions and proposed structure.

Expected Fragmentions	Sum formula	m/z
y1	$C_{28}H_{63}N_4O$	471.4996
y2	$C_{37}H_{78}N_5O_4$	656.6048
y5	$C_{49}H_{95}N_{10}O_9$	967.7278
y6	$C_{55}H_{102}N_{13}O_{10}$	1104.7867
b1	$C_4H_6NO_3$	116.0342
b2	$C_{10}H_{13}N_4O_4$	253.0931
b5	$C_{22}H_{30}N_9O_9$	564.2161
b6	$C_{31}H_{45}N_{10}O_{12}$	749.3213

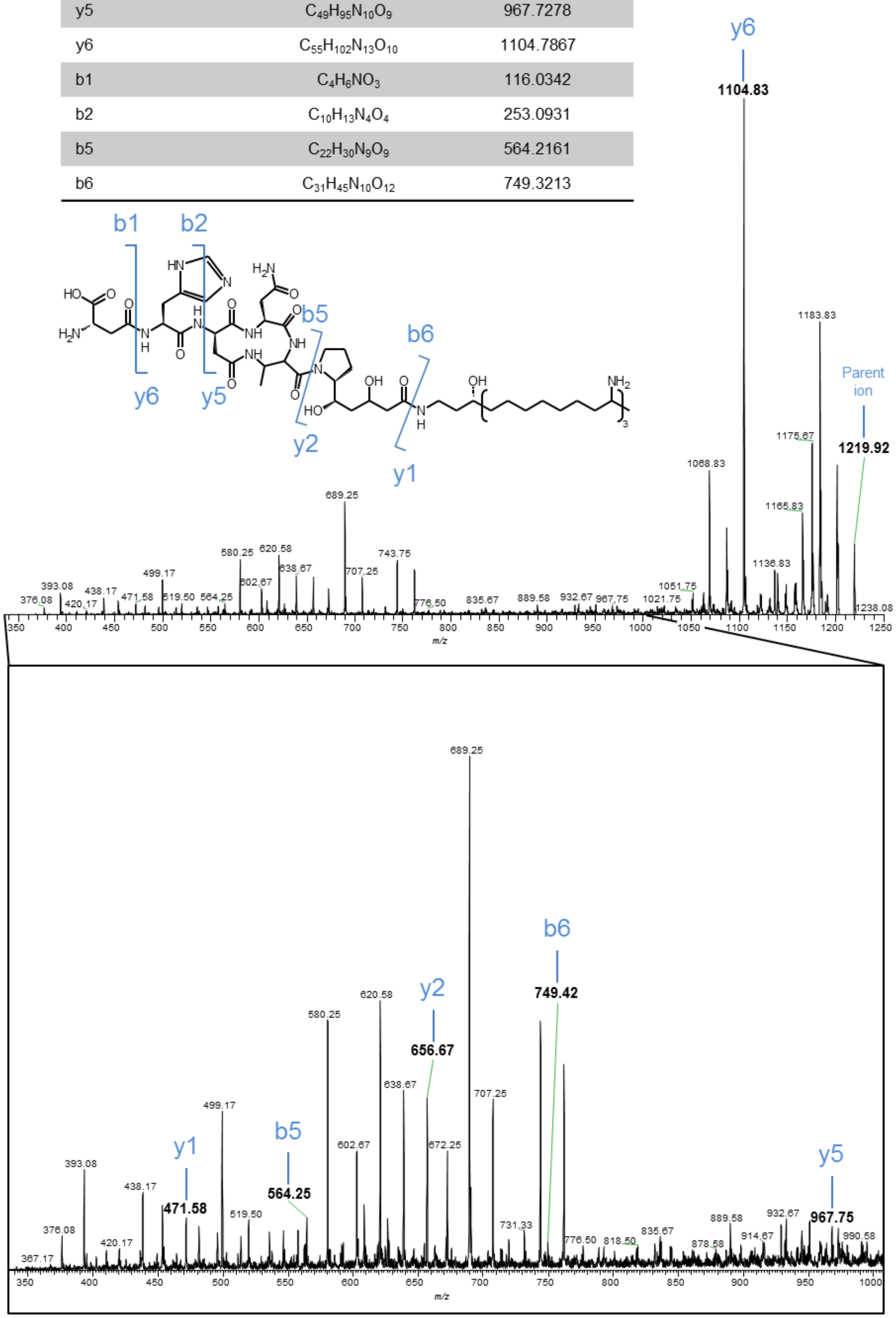

Figure S10: MALDI–MS^2 spectra of compound 1219.81 (**6**) of *X. budapestensis* pCEP_*fcl* (induced) showing expected fragment ions and proposed structure.

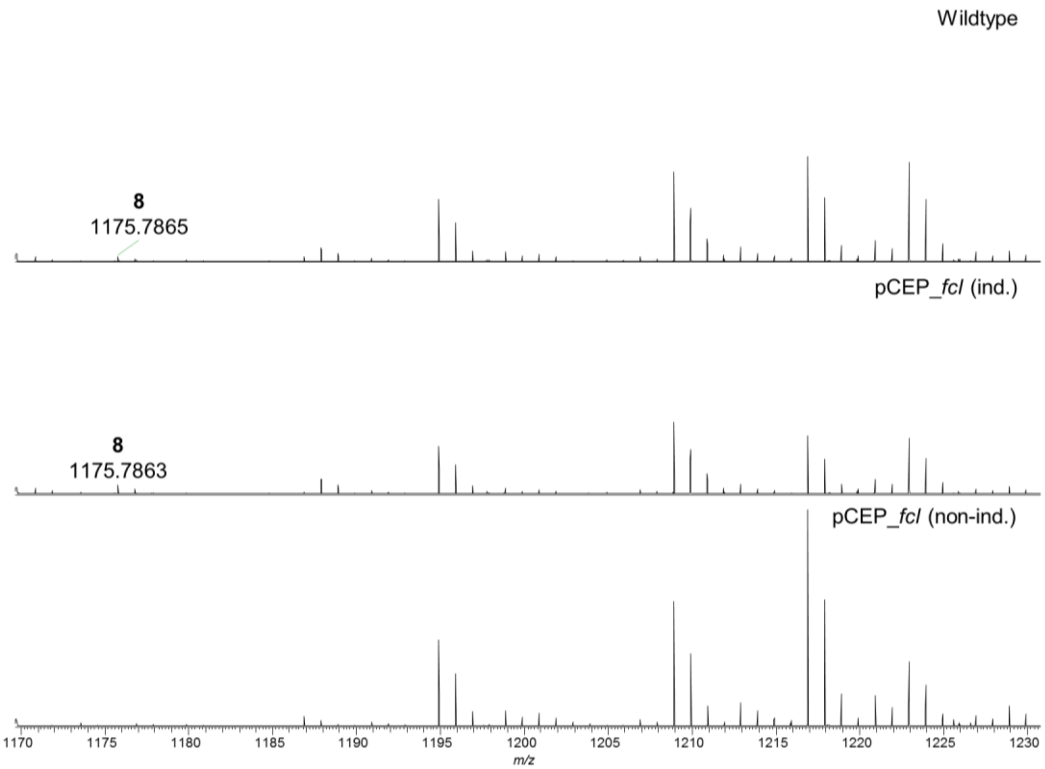

#	Sum formula	Calc. m/z	Det. m/z		Δppm	
			WT	pCEP_*fcl*	WT	pCEP_*fcl*
**8**	$C_{57}H_{102}N_{14}O_{12}$	1175.7874	1175.7865	1175.7863	-0.834	-1.004
**7**	$C_{60}H_{104}N_{12}O_{12}$	1185.7969	1185.7952	1185.7947	-1.495	-1.892
**6**	$C_{59}H_{106}N_{14}O_{13}$	1219.8137	1219.8132	1219.8129	-0.398	-0.652
**5**	$C_{62}H_{108}N_{12}O_{13}$	1229.8232	1229.8221	1229.8229	-0.860	-0.234

Figure S11: MALDI–HRMS spectra of *X. indica* wild type (WT) and pCEP_*fcl* promoter-exchange mutant (induced and noninduced) with compounds **5**–**8** showing sum formulas, calculated and detected masses and corresponding Δppm. Due to unspecific signals in all three spectra with high intensities the compounds **5**–**7** are not visible, but could be confirmed by HRMS.

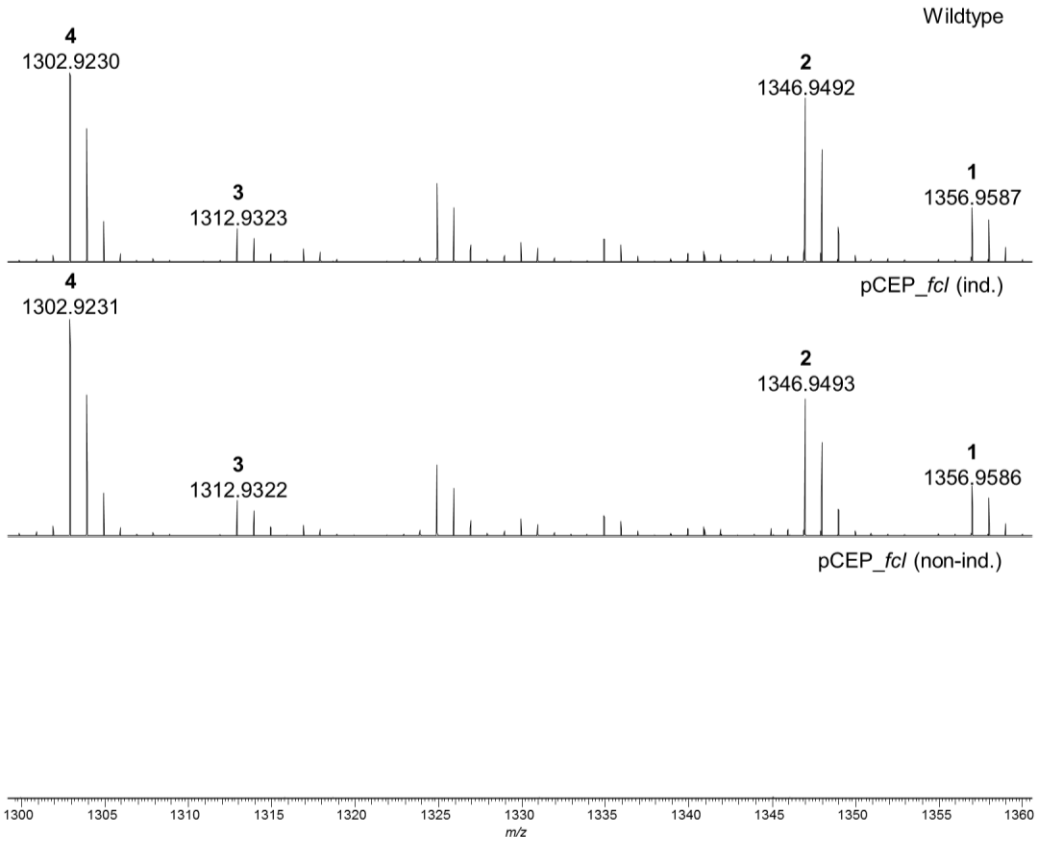

#	Sum formula	Calc. m/z	Det. m/z		Δppm	
			WT	pCEP_*fcl*	WT	pCEP_*fcl*
**4**	$C_{65}H_{119}N_{15}O_{12}$	1302.9235	1302.9230	1302.9231	-0.453	-0.361
**3**	$C_{68}H_{121}N_{13}O_{12}$	1312.9330	1312.9323	1312.9322	-0.558	-0.626
**2**	$C_{67}H_{123}N_{15}O_{13}$	1346.9498	1346.9492	1346.9493	-0.397	-0.323
**1**	$C_{70}H_{125}N_{13}O_{13}$	1356.9593	1356.9587	1356.9586	-0.447	-0.492

Figure S12: MALDI–HRMS spectra of *X. indica* wild type (WT) and pCEP_*fcl* promoter-exchange mutant (induced and noninduced) with compounds **1–4** showing sum formulas, calculated and detected masses and corresponding Δppm.

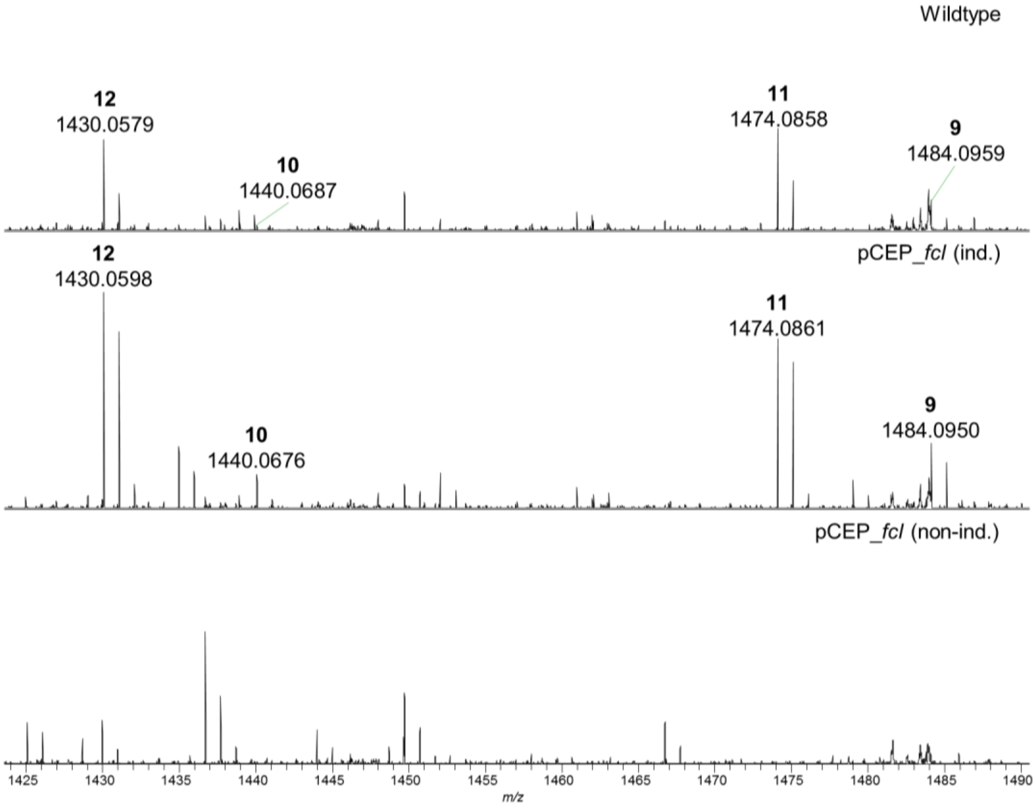

#	Sum formula	Calc. m/z	Det. m/z		Δppm	
			WT	pCEP_*fcl*	WT	pCEP_*fcl*
**12**	$C_{73}H_{136}N_{16}O_{12}$	1430.0596	1430.0579	1430.0598	-1.238	0.084
**10**	$C_{76}H_{138}N_{14}O_{12}$	1440.0691	1440.0687	1440.0676	-0.286	-1.043
**11**	$C_{75}H_{140}N_{16}O_{13}$	1474.0859	1474.0858	1474.0861	-0.030	0.180
**9**	$C_{78}H_{142}N_{14}O_{13}$	1484.0954	1484.0959	1484.0951	0.359	-0.207

Figure S13: MALDI–HRMS spectra of *X. indica* wild type (WT) and pCEP_*fcl* promoter-exchange mutant (induced and noninduced) with compounds **9**–**12** showing sum formulas, calculated and detected masses and corresponding Δppm.

Expected Fragmentions	Sum formula	m/z
y1	$C_{44}H_{97}N_6O$	725.7718
y2	$C_{51}H_{108}N_7O_3$	866.8508
y5	$C_{63}H_{125}N_{12}O_8$	1177.9738
y6	$C_{69}H_{132}N_{15}O_9$	1315.0327
b1	$C_4H_6NO_3$	116.0342
b2	$C_{10}H_{13}N_4O_4$	253.0931
b5	$C_{22}H_{30}N_9O_9$	564.2161
b6	$C_{29}H_{41}N_{10}O_{11}$	705.2951

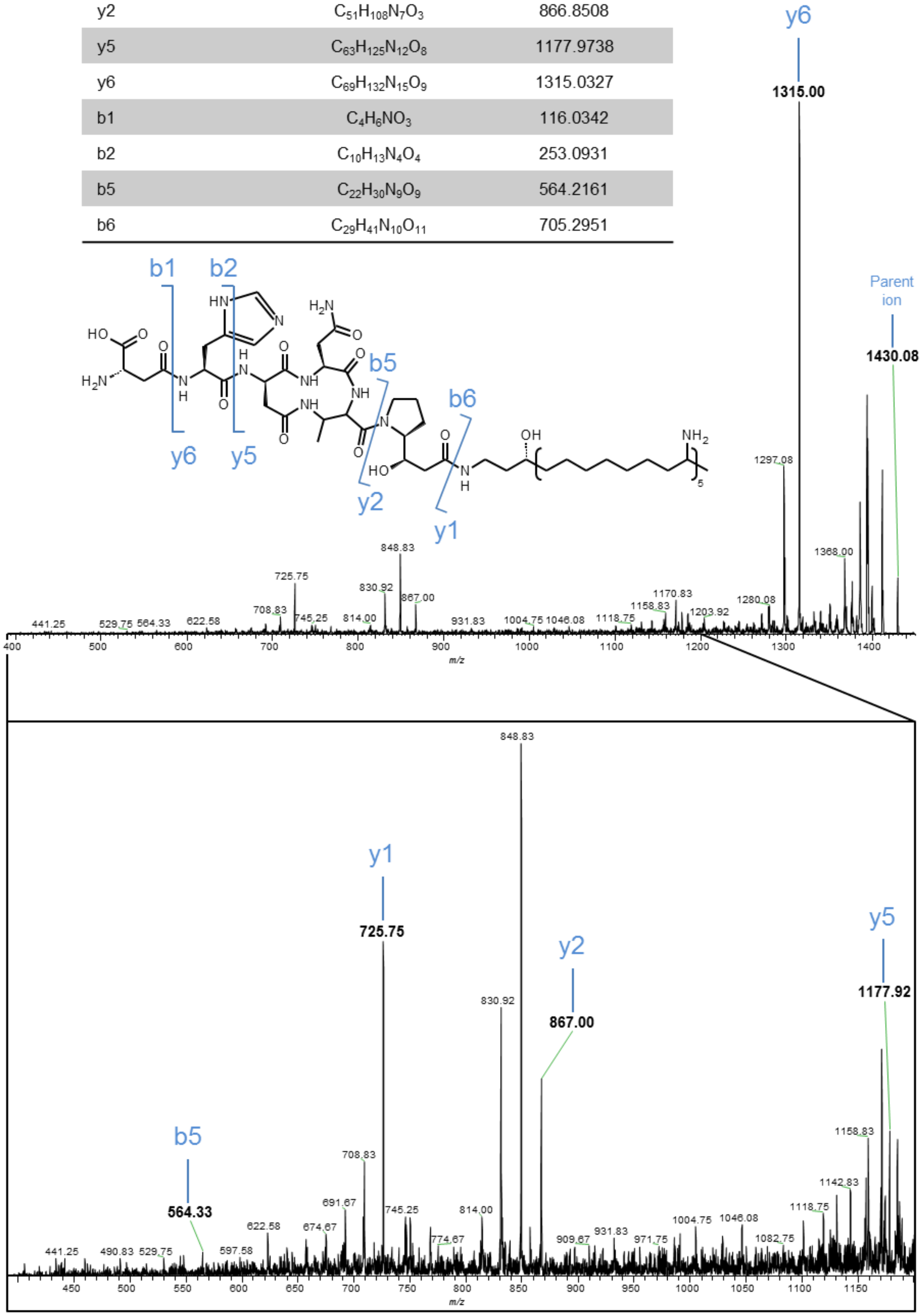

Figure S14: MALDI–MS^2 spectra of compound 1430.05 (**12**) of *X. indica* pCEP_*fcl* (induced) showing expected fragment ions and proposed structure.

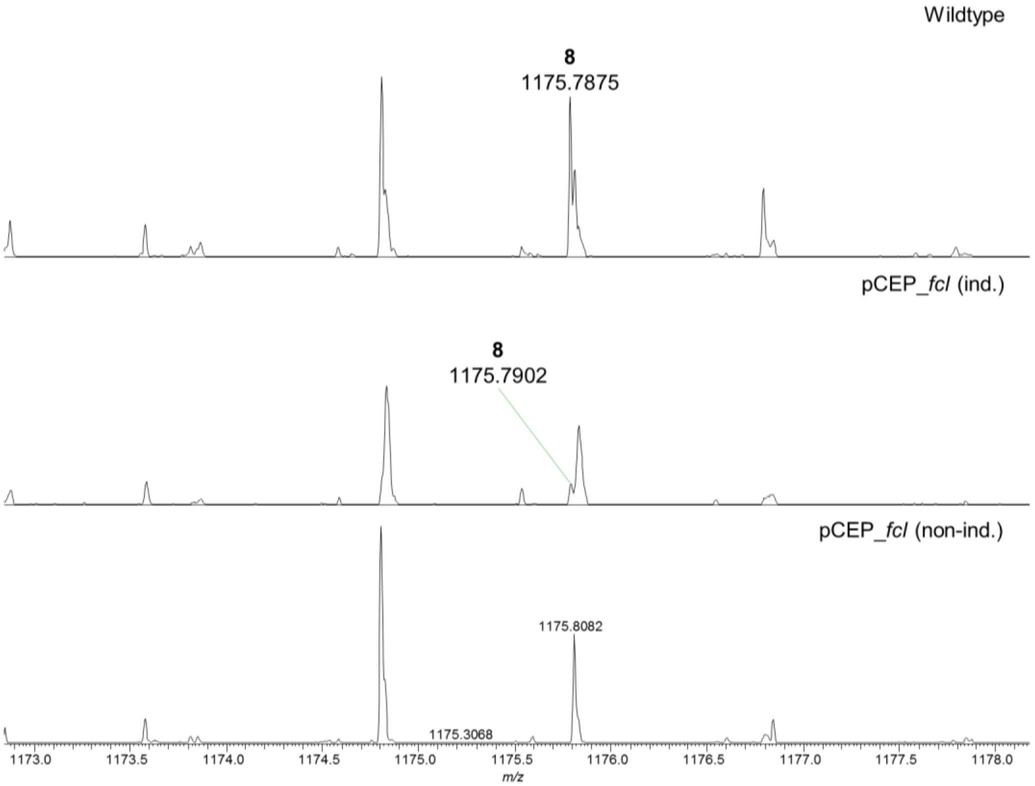

#	Sum formula	Calc. m/z	Det. m/z		Δppm	
			WT	pCEP_*fcl*	WT	pCEP_*fcl*
**8**	$C_{57}H_{102}N_{14}O_{12}$	1175.7874	1175.7875	1175.7902	0.050	2.355

Figure S15: MALDI–HRMS spectra of *X. cabanillasii* wild type (WT) and pCEP_*fcl* promoter-exchange mutant (induced and noninduced) with compound **8** showing sum formula, calculated and detected masses and corresponding Δppm.

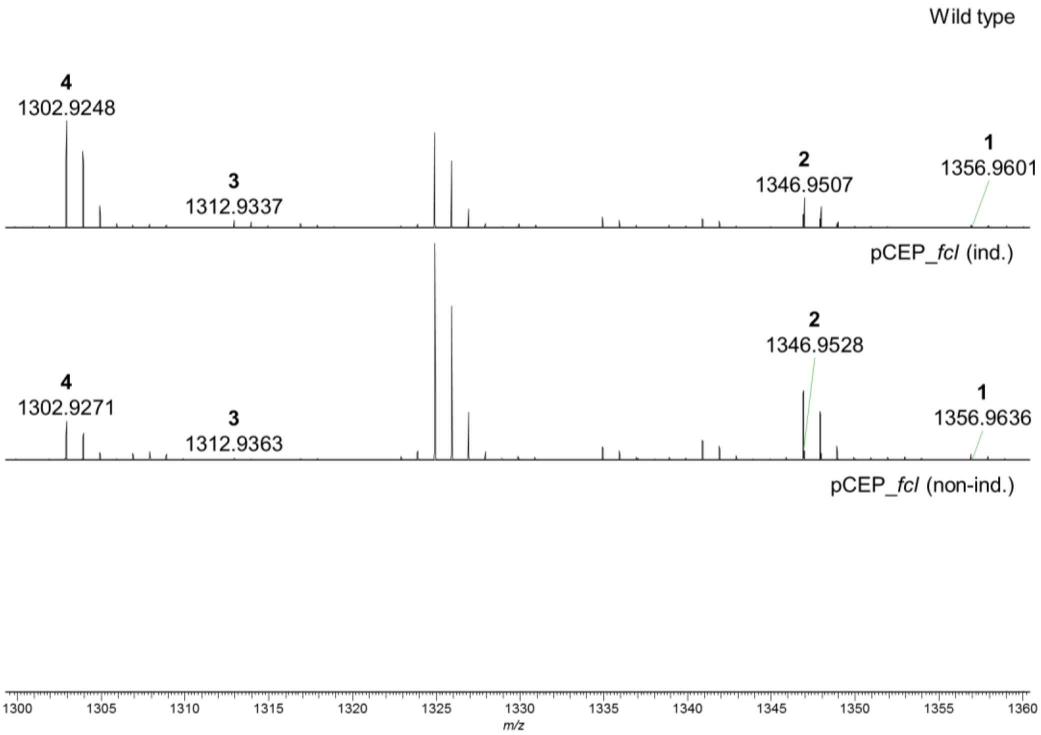

#	Sum formula	Calc. m/z	Det. m/z		Δppm	
			WT	pCEP_*fcl*	WT	pCEP_*fcl*
4	$C_{65}H_{119}N_{15}O_{12}$	1302.9235	1302.9248	1302.9271	0.990	2.747
3	$C_{68}H_{121}N_{13}O_{12}$	1312.9330	1312.9337	1312.9363	0.516	2.481
2	$C_{67}H_{123}N_{15}O_{13}$	1346.9498	1346.9507	1346.9528	0.679	2.275
1	$C_{70}H_{125}N_{13}O_{13}$	1356.9593	1356.9601	1356.9636	0.636	3.193

Figure S16: MALDI–HRMS spectra of *X. cabanillasii* wild type (WT) and pCEP_*fcl* promoter-exchange mutant (induced and noninduced) with compounds **1**–**4** showing sum formulas, calculated and detected masses and corresponding Δppm. Compounds **1**–**4** were described previously [14].

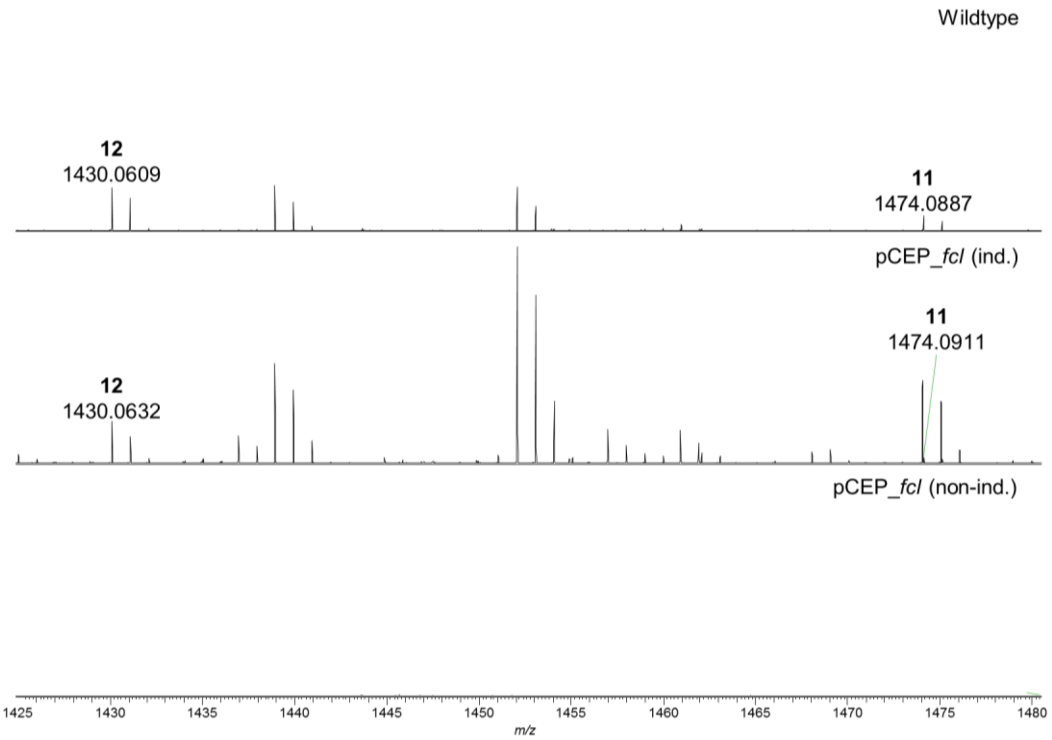

#	Sum formula	Calc. m/z	Det. m/z		Δppm	
			WT	pCEP_*fcl*	WT	pCEP_*fcl*
**12**	$C_{73}H_{136}N_{16}O_{12}$	1430.0596	1430.0609	1430.0633	0.860	2.524
**11**	$C_{75}H_{140}N_{16}O_{13}$	1474.0859	1474.0887	1474.0911	1.930	3.586

Figure S17: MALDI–HRMS spectra of *X. cabanillasii* wild type (WT) and pCEP_*fcl* promoter-exchange mutant (induced and noninduced) with compounds **11** and **12** showing sum formulas, calculated and detected masses and corresponding Δppm.

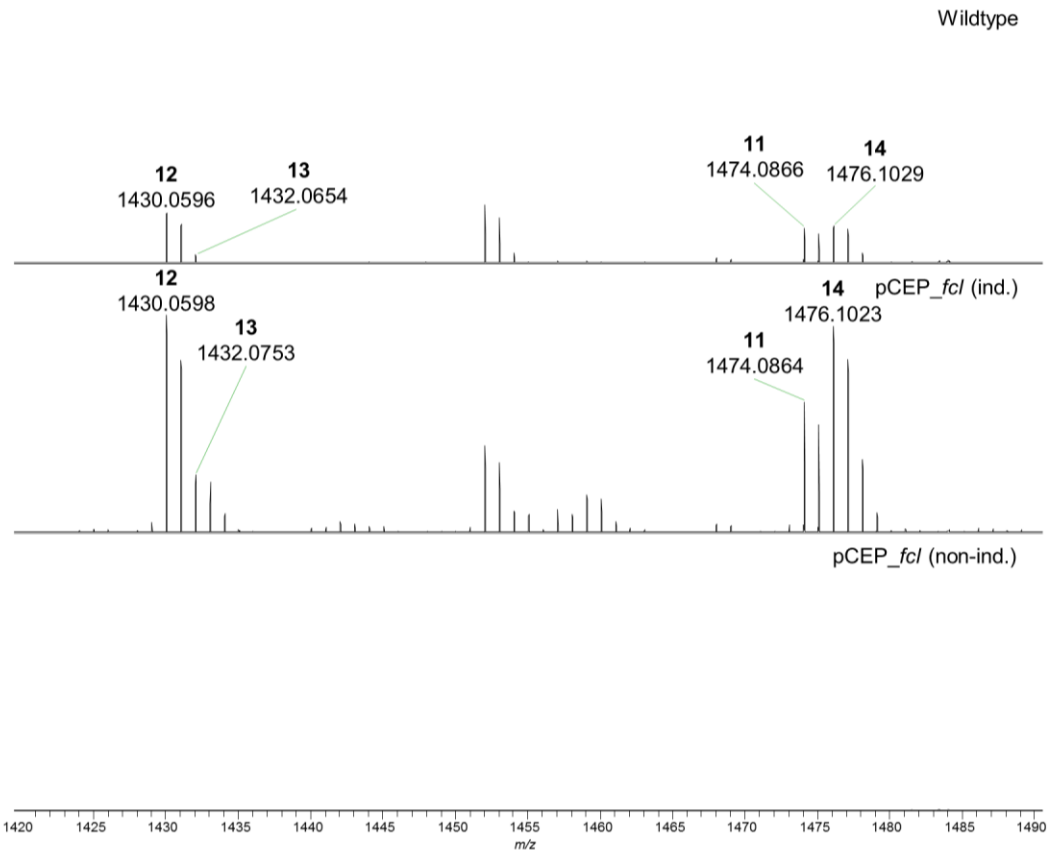

#	Sum formula	Calc. m/z	Det. m/z		Δppm	
			WT	pCEP_*fcl*	WT	pCEP_*fcl*
**12**	$C_{73}H_{136}N_{16}O_{12}$	1430.0596	1430.0596	1430.0599	0.000	0.147
**13**	$C_{73}H_{138}N_{16}O_{12}$	1432.0753	1432.0654	1432.0753	-6.913	0.021
**11**	$C_{75}H_{140}N_{16}O_{13}$	1474.0859	1474.0866	1474.0864	0.485	0.370
**14**	$C_{75}H_{142}N_{16}O_{13}$	1476.1015	1476.1029	1476.1023	0.972	0.532

Figure S18: MALDI–HRMS spectra of *X. hominickii* wild type (WT) and pCEP_*fcl* promoter-exchange mutant (induced and noninduced) with compounds **11–14** showing sum formulas, calculated and detected masses and corresponding Δppm.

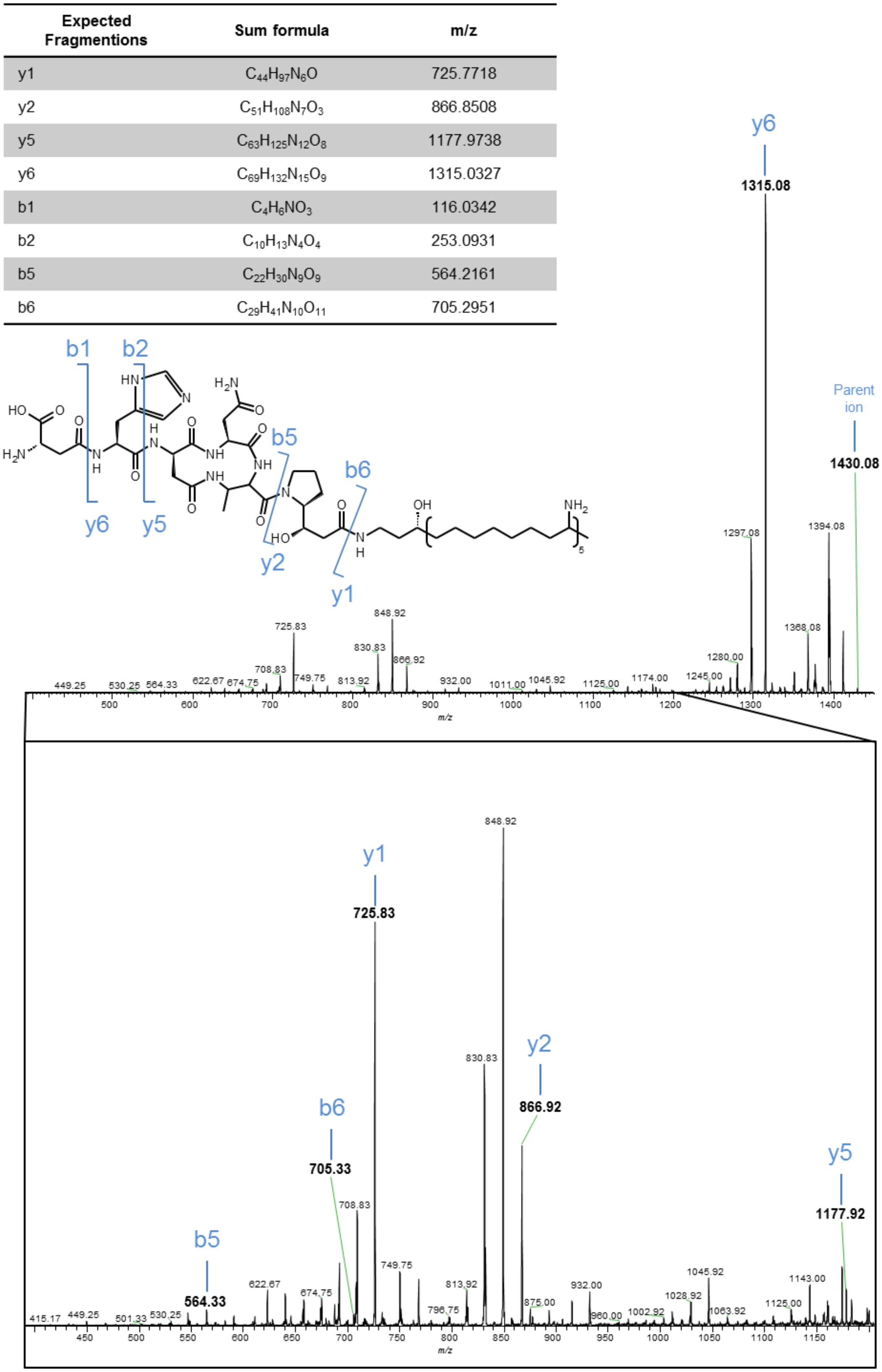

Expected Fragmentions	Sum formula	m/z
y1	$C_{44}H_{97}N_6O$	725.7718
y2	$C_{51}H_{108}N_7O_3$	866.8508
y5	$C_{63}H_{125}N_{12}O_8$	1177.9738
y6	$C_{69}H_{132}N_{15}O_9$	1315.0327
b1	$C_4H_6NO_3$	116.0342
b2	$C_{10}H_{13}N_4O_4$	253.0931
b5	$C_{22}H_{30}N_9O_9$	564.2161
b6	$C_{29}H_{41}N_{10}O_{11}$	705.2951

Figure S19: MALDI–MS^2 spectra of compound 1430.05 (**12**) of *X. hominickii* pCEP_*fcl* (induced) showing expected fragment ions and proposed structure.

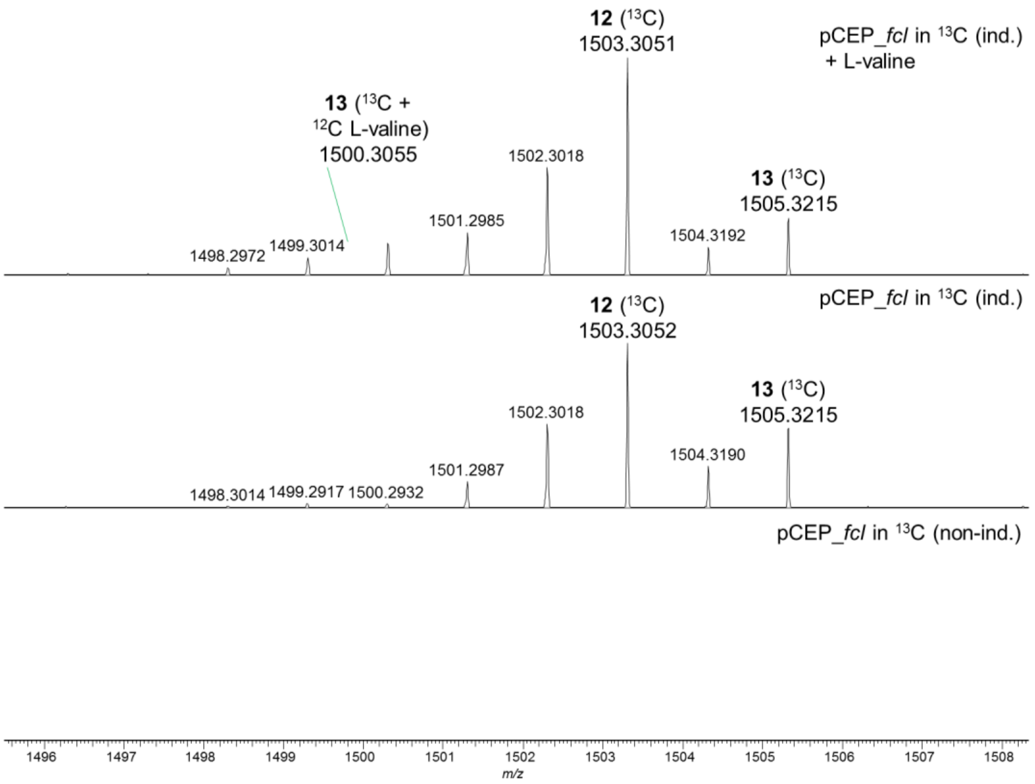

#	Sum formula	Calc. m/z	Det. m/z		Δppm	
			pCEP_*fcl*	pCEP_*fcl* + L-valine	pCEP_*fcl*	pCEP_*fcl* + L-valine
**12** (^{13}C)	$^{13}C_{73}H_{136}N_{16}O_{12}$	1503.3048	1503.3052	1503.3051	0.2727	0.2262
**13** (^{13}C)	$^{13}C_{73}H_{138}N_{16}O_{12}$	1505.3204	1505.3215	1505.3215	0.6842	0.7241
**13** (^{13}C + ^{12}C L-valine)	$^{13}C_{68}C_{5}H_{138}N_{16}O_{12}$	1500.3037	-	1500.3055	-	1.2131

Figure S20: MALDI–HRMS spectra of the reverse feeding experiment in ^{13}C medium of *X. hominickii* pCEP_*fcl* mutant (induced and noninduced) with compounds **12** and **13** (with and without feeding of ^{12}C L-valine) showing sum formulas, calculated and detected masses and corresponding Δppm.

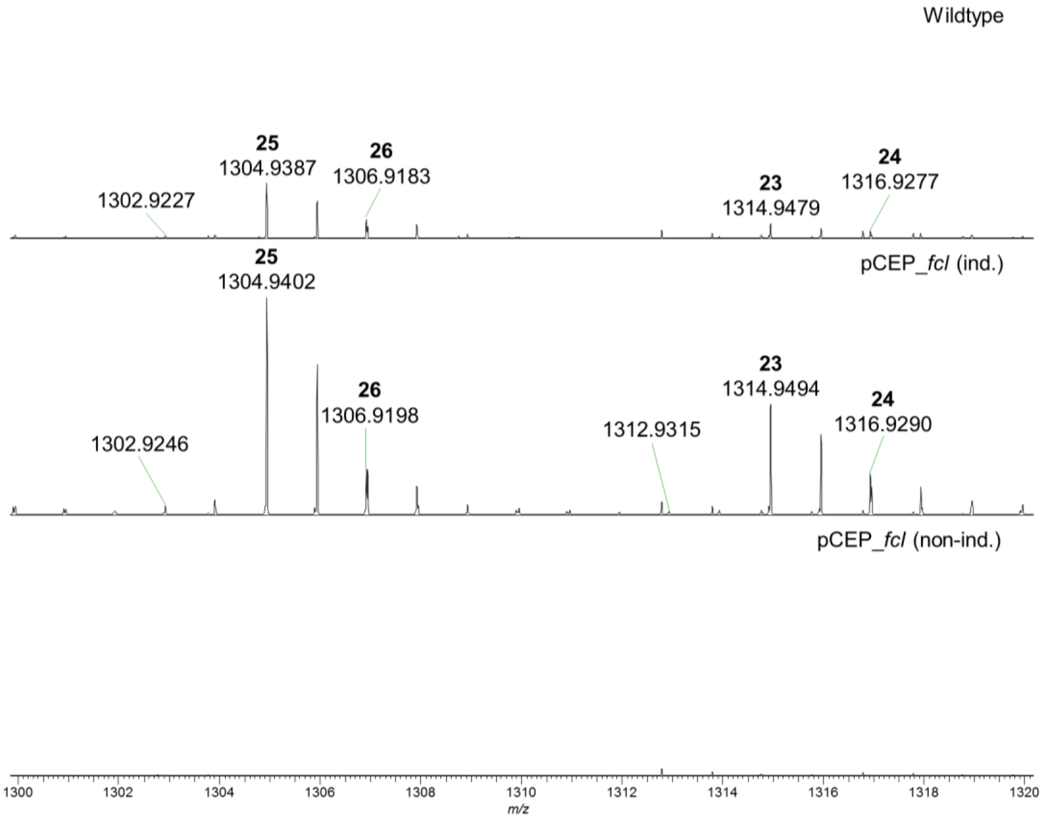

#	Sum formula	Calc. m/z	Det. m/z		Δppm	
			WT	pCEP_*fcl*	WT	pCEP_*fcl*
-	$C_{65}H_{119}N_{15}O_{12}$	1302.9235	1302.9227	1302.9246	-0.630	0.813
**25**	$C_{65}H_{121}N_{15}O_{12}$	1304.9392	1304.9387	1304.9402	-0.384	0.774
**26**	$C_{64}H_{119}N_{15}O_{13}$	1306.9185	1306.9183	1306.9198	-0.149	1.029
-	$C_{68}H_{121}N_{13}O_{12}$	1312.9330	1312.9290	1312.9315	-3.064	-1.167
**23**	$C_{68}H_{123}N_{13}O_{12}$	1314.9487	1314.9479	1314.9494	-0.595	0.568
**24**	$C_{67}H_{121}N_{13}O_{13}$	1316.9280	1316.9277	1316.9290	-0.218	0.769

Figure S21: MALDI–HRMS spectra of KJ12.1 wild type (WT) and pCEP_*fcl* promoter-exchange mutant (induced and noninduced) with compounds **23**–**26** showing sum formulas, calculated and detected masses and corresponding Δppm.

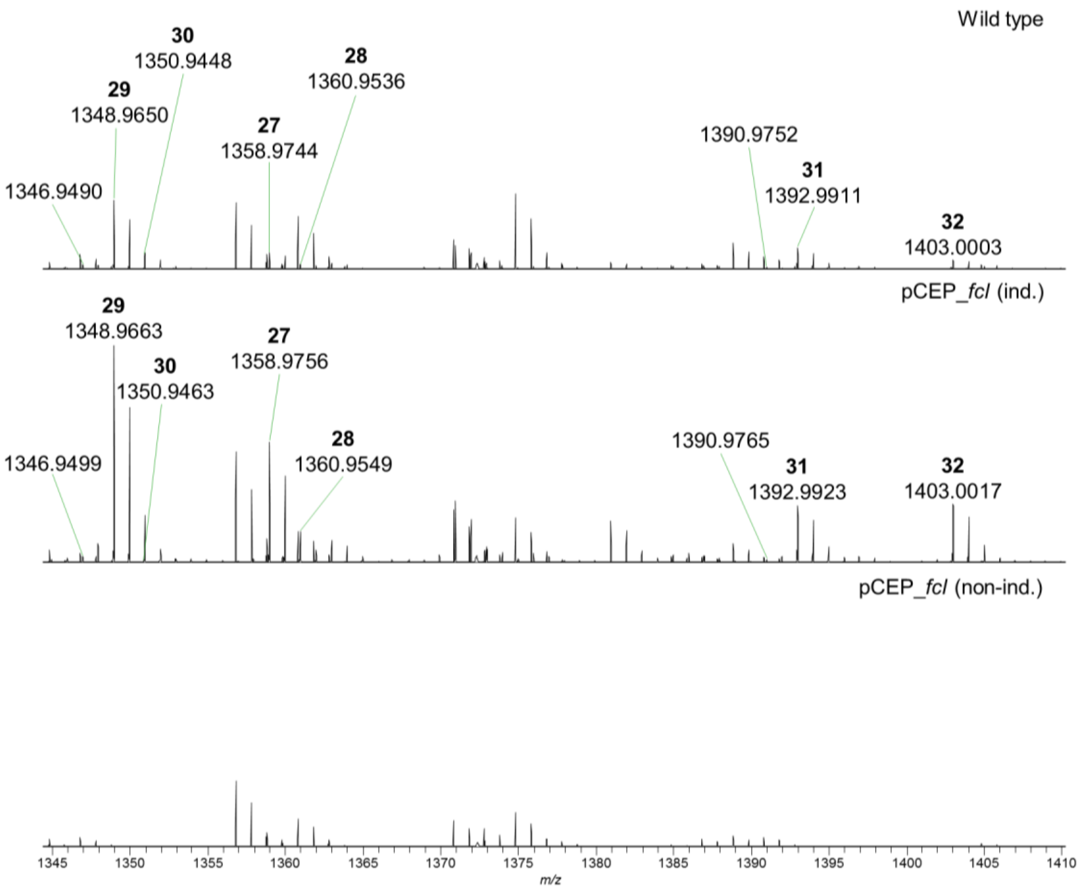

#	Sum formula	Calc. m/z	Det. m/z		Δppm	
			WT	pCEP_*fcl*	WT	pCEP_*fcl*
-	$C_{67}H_{123}N_{15}O_{13}$	1346.9498	1346.9490	1346.9499	-0.575	0.115
**29**	$C_{67}H_{125}N_{15}O_{13}$	1348.9654	1348.9650	1348.9663	-0.323	0.626
**30**	$C_{66}H_{123}N_{15}O_{14}$	1350.9447	1350.9448	1350.9463	0.096	1.222
**27**	$C_{70}H_{127}N_{13}O_{13}$	1358.9749	1358.9744	1358.9756	-0.403	0.480
**28**	$C_{69}H_{125}N_{13}O_{14}$	1360.9542	1360.9536	1360.9549	-0.442	0.542
-	$C_{69}H_{127}N_{15}O_{14}$	1390.9760	1390.9752	1390.9765	-0.546	0.403
**31**	$C_{69}H_{129}N_{15}O_{14}$	1392.9916	1392.9911	1392.9923	-0.366	0.467
**32**	$C_{72}H_{131}N_{13}O_{14}$	1403.0011	1403.0003	1403.0017	-0.593	0.398

Figure S22: MALDI–HRMS spectra of KJ12.1 wild type (WT) and pCEP_*fcl* promoter-exchange mutant (induced and noninduced) with compounds **27–32** showing sum formulas, calculated and detected masses and corresponding Δppm.

Expected Fragmentions	Sum formula	m/z
y1	$C_{36}H_{80}N_5O$	598.6357
y2	$C_{43}H_{93}N_6O_3$	741.7304
y5	$C_{55}H_{110}N_{11}O_8$	1052.8533
y6	$C_{61}H_{117}N_{14}O_9$	1189.9122
b1	$C_4H_6NO_3$	116.0342
b2	$C_{10}H_{13}N_4O_4$	253.0931
b5	$C_{22}H_{30}N_9O_9$	564.2161
b6	$C_{29}H_{43}N_{10}O_{11}$	707.3107

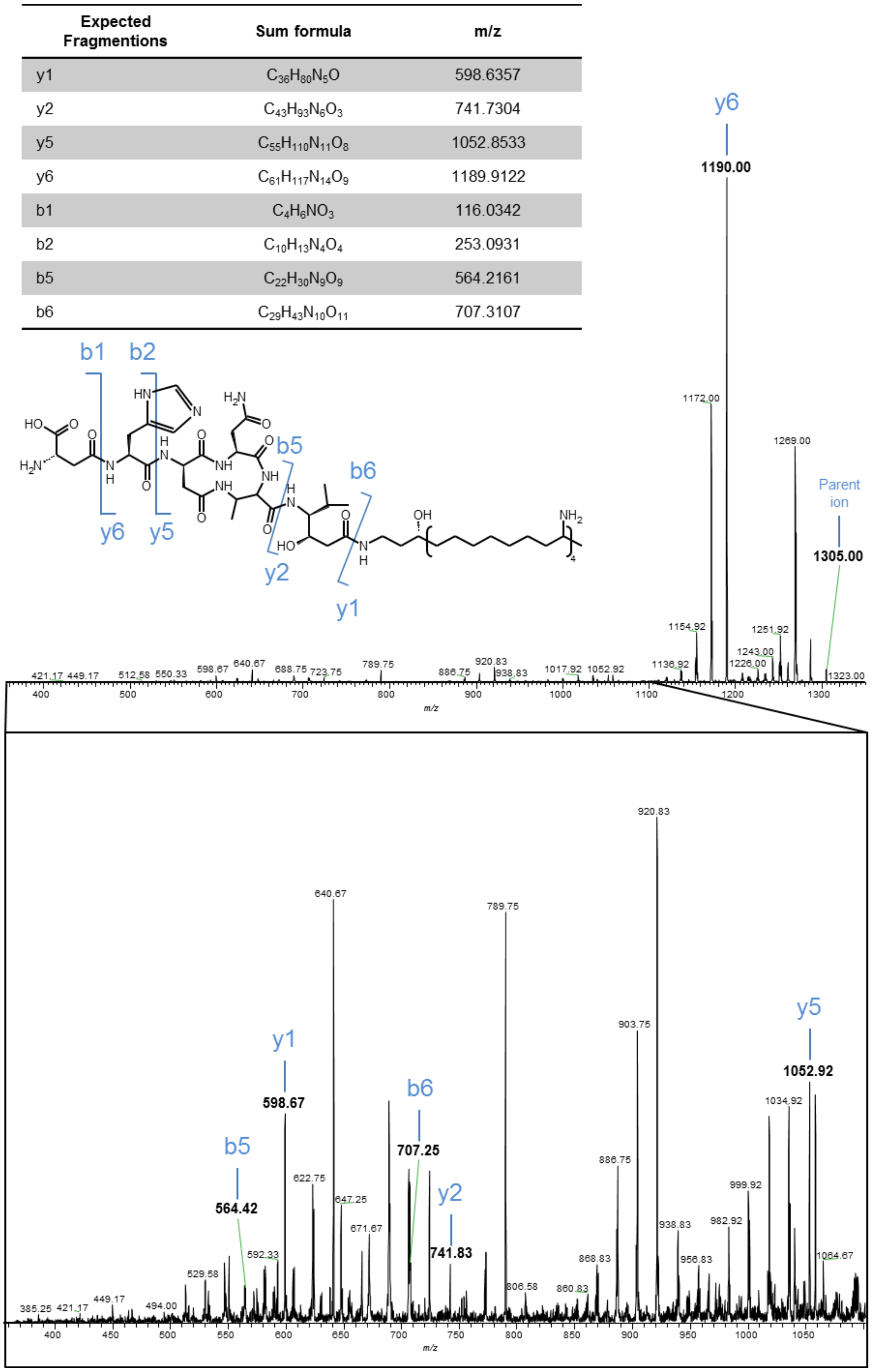

Figure S23: MALDI–MS^2 spectra of compound 1304.94 (**25**) of KJ12.1 pCEP_*fcl* (induced) showing expected fragment ions and proposed structure.

Expected Fragmentions	Sum formula	m/z
y1	$C_{36}H_{80}N_5O$	598.6357
y2	$C_{42}H_{91}N_6O_4$	743.7096
y5	$C_{54}H_{108}N_{11}O_9$	1054.8326
y6	$C_{60}H_{115}N_{14}O_{10}$	1191.8915
b1	$C_4H_6NO_3$	116.0342
b2	$C_{10}H_{13}N_4O_4$	253.0931
b5	$C_{22}H_{30}N_9O_9$	564.2161
b6	$C_{28}H_{41}N_{10}O_{12}$	709.29

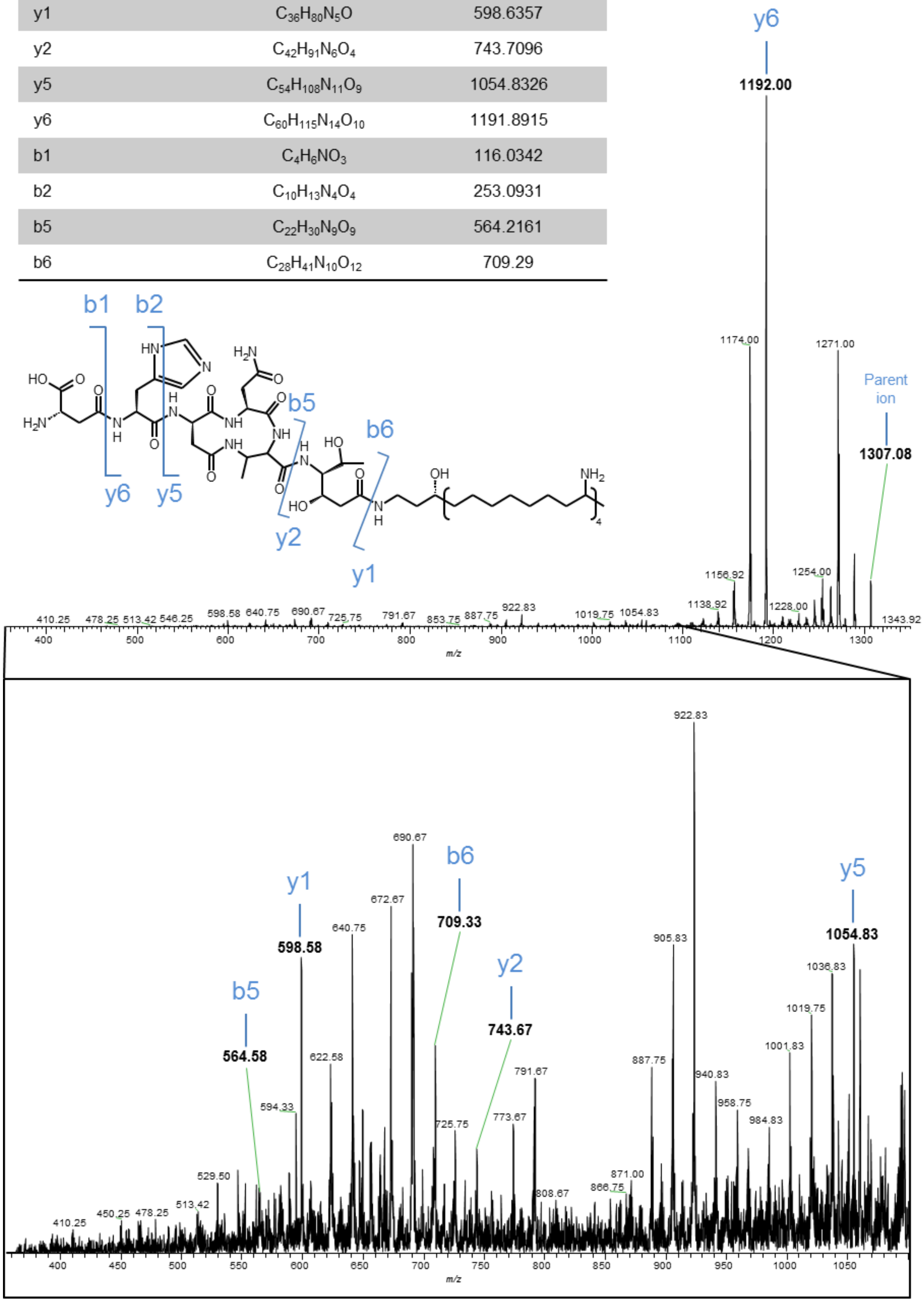

Figure S24: MALDI–MS^2 spectra of compound 1306.91 (**26**) of KJ12.1 pCEP_*fcl* (induced) showing expected fragment ions and proposed structure.

Expected Fragmentions	Sum formula	m/z
y1	$C_{36}H_{80}N_5O$	598.6357
y2	$C_{47}H_{101}N_6O_5$	829.7828
y5	$C_{59}H_{118}N_{11}O_{10}$	1140.9058
y6	$C_{65}H_{125}N_{14}O_{11}$	1277.9647
b1	$C_4H_6NO_3$	116.0342
b2	$C_{10}H_{13}N_4O_4$	253.0931
b5	$C_{22}H_{30}N_9O_9$	564.2161
b6	$C_{33}H_{51}N_{10}O_{13}$	795.3632

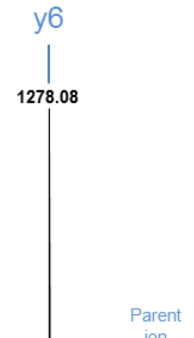

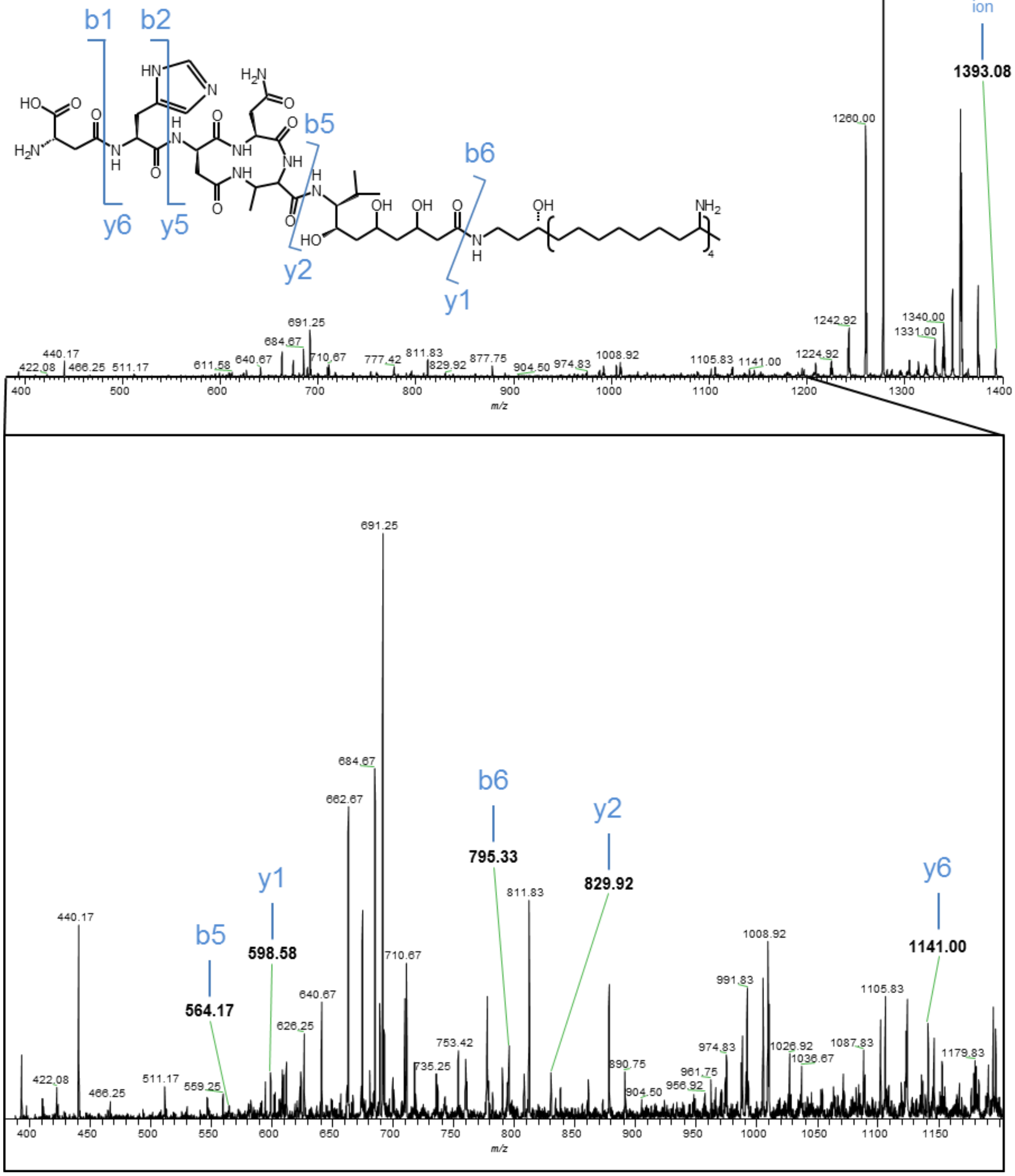

Figure S25: MALDI–MS^2 spectra of compound 1392.99 (**31**) of KJ12.1 pCEP_*fcl* (induced) showing expected fragment ions and proposed structure.

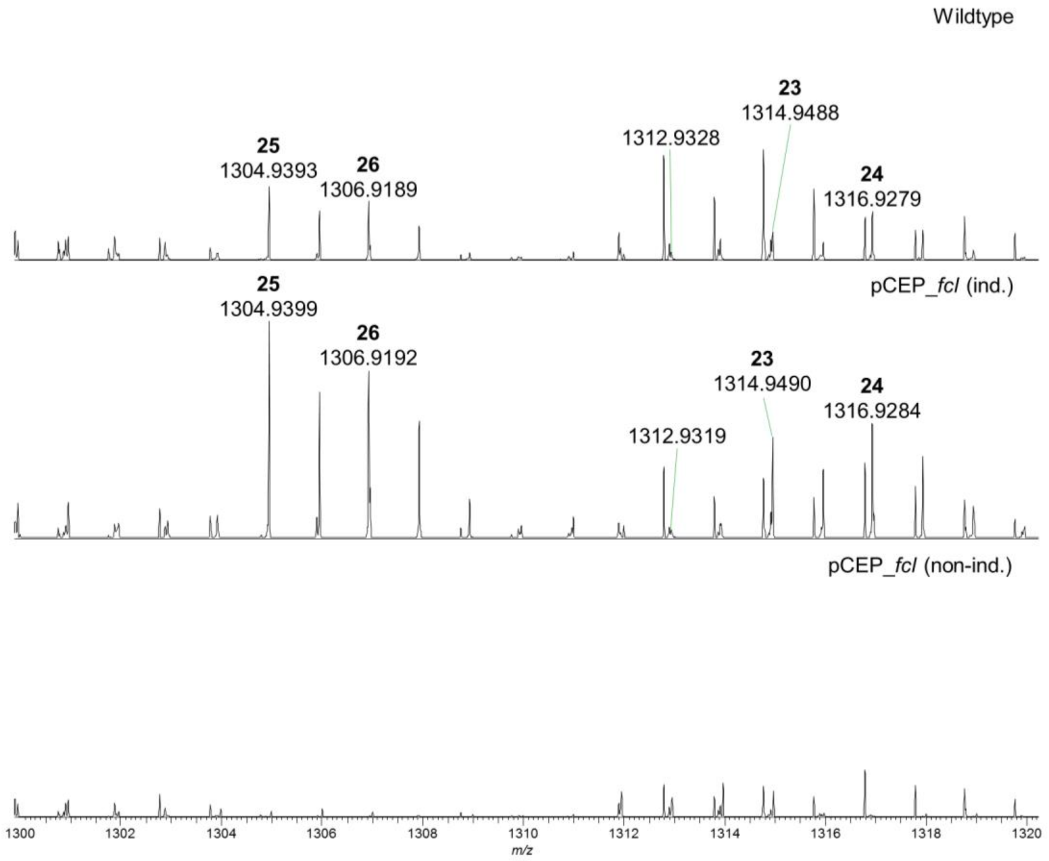

#	Sum formula	Calc. m/z	Det. m/z		Δppm	
			WT	pCEP_*fcl*	WT	pCEP_*fcl*
**25**	$C_{65}H_{121}N_{15}O_{12}$	1304.9392	1304.9393	1304.9399	0.046	0.521
**26**	$C_{64}H_{119}N_{15}O_{13}$	1306.9185	1306.9189	1306.9192	0.371	0.570
-	$C_{68}H_{121}N_{13}O_{12}$	1312.9330	1312.9328	1312.9319	-0.192	-0.870
**23**	$C_{68}H_{123}N_{13}O_{12}$	1314.9487	1314.9488	1314.9490	0.112	0.241
**24**	$C_{67}H_{121}N_{13}O_{13}$	1316.9280	1316.9279	1316.9284	-0.066	0.359

Figure S26: MALDI–HRMS spectra of *X. stockiae* wild type (WT) and pCEP_*fcl* promoter-exchange mutant (induced and noninduced) with compounds **23**–**26** showing sum formulas, calculated and detected masses and corresponding Δppm.

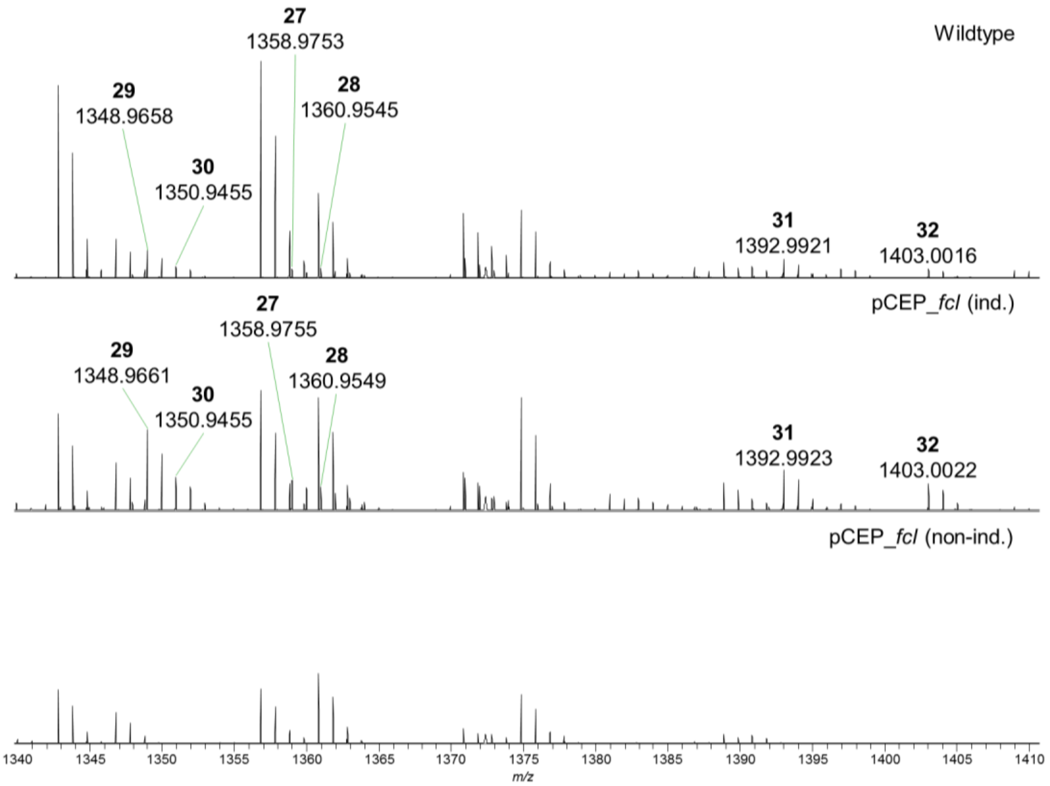

#	Sum formula	Calc. m/z	Det. m/z		Δppm	
			WT	pCEP_*fcl*	WT	pCEP_*fcl*
**29**	$C_{67}H_{125}N_{15}O_{13}$	1348.9654	1348.9658	1348.9661	0.285	0.545
**30**	$C_{66}H_{123}N_{15}O_{14}$	1350.9447	1350.9455	1350.9455	0.615	0.629
**27**	$C_{70}H_{127}N_{13}O_{13}$	1358.9749	1358.9753	1358.9755	0.318	0.429
**28**	$C_{69}H_{125}N_{13}O_{14}$	1360.9542	1360.9545	1360.9549	0.226	0.520
**31**	$C_{69}H_{129}N_{15}O_{14}$	1392.9916	1392.9922	1392.9923	0.381	0.467
**32**	$C_{72}H_{131}N_{13}O_{14}$	1403.0011	1403.0016	1403.0022	0.334	0.747

Figure S27: MALDI–HRMS spectra of *X. stockiae* wild type (WT) and pCEP_*fcl* promoter-exchange mutant (induced and-noninduced) with compounds **27**–**32** showing sum formulas, calculated and detected masses and corresponding Δppm.

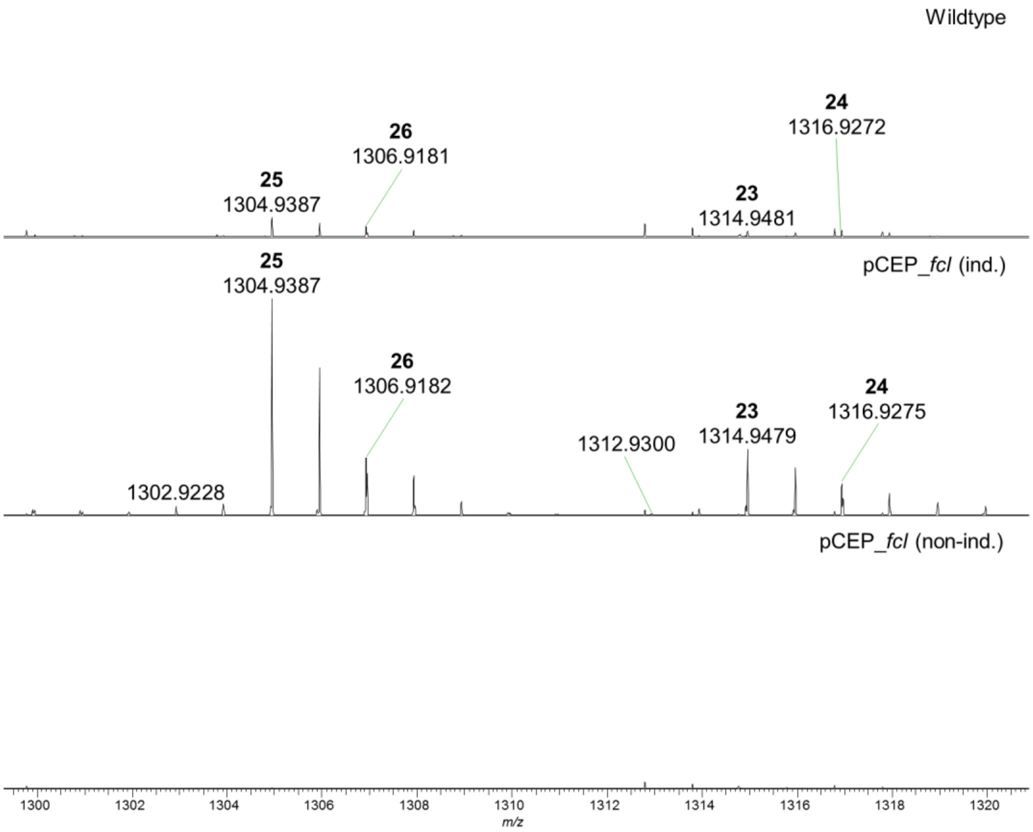

#	Sum formula	Calc. m/z	Det. m/z		Δppm	
			WT	pCEP_*fcl*	WT	pCEP_*fcl*
-	$C_{65}H_{119}N_{15}O_{12}$	1302.9235	1302.9242	1302.9228	0.499	-0.561
**25**	$C_{65}H_{121}N_{15}O_{12}$	1304.9392	1304.9387	1304.9387	-0.407	-0.361
**26**	$C_{64}H_{119}N_{15}O_{13}$	1306.9185	1306.9181	1306.9182	-0.279	-0.172
-	$C_{68}H_{121}N_{13}O_{12}$	1312.9330	1312.9287	1312.9300	-3.285	-2.317
**23**	$C_{68}H_{123}N_{13}O_{12}$	1314.9487	1314.9481	1314.9479	-0.489	-0.587
**24**	$C_{67}H_{121}N_{13}O_{13}$	1316.928	1316.9272	1316.9275	-0.544	-0.324

Figure S28: MALDI–HRMS spectra of KK7.4 wild type (WT) and pCEP_*fcl* promoter-exchange mutant (induced and noninduced) with compounds **23**–**26** showing sum formulas, calculated and detected masses and corresponding Δppm.

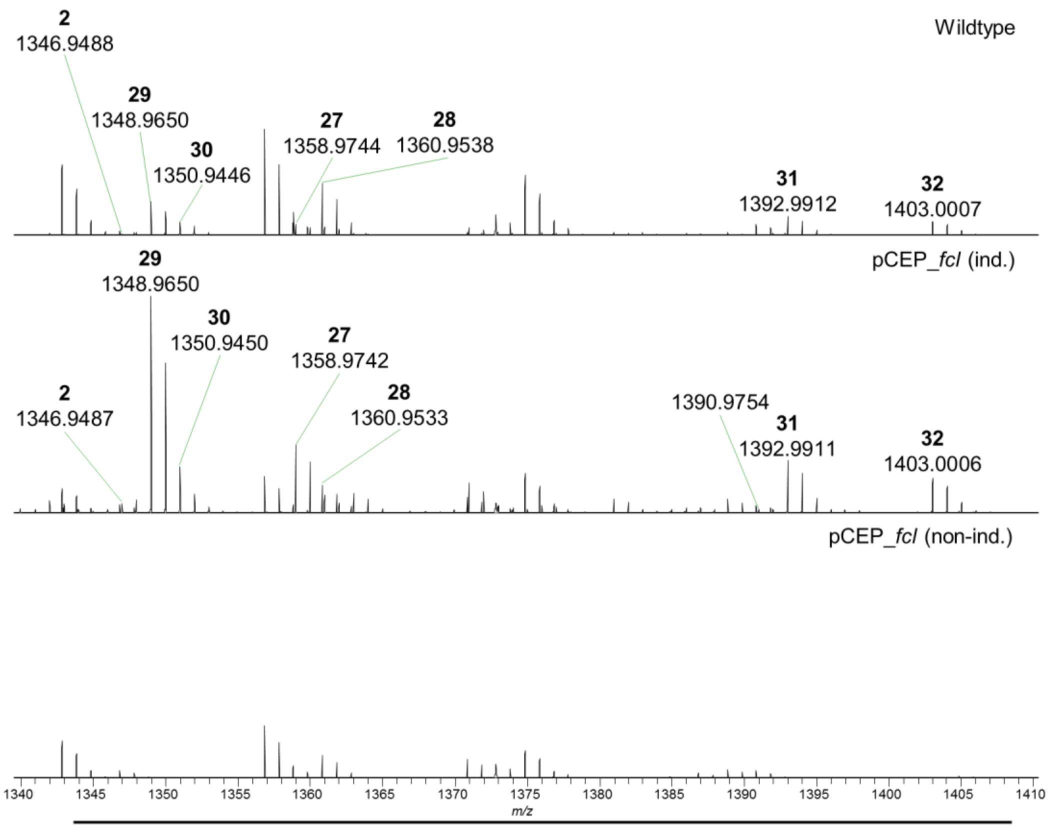

#	Sum formula	Calc. m/z	Det. m/z		Δppm	
			WT	pCEP_*fcl*	WT	pCEP_*fcl*
**2**	$C_{67}H_{123}N_{15}O_{13}$	1346.9498	1346.9488	1346.9487	-0.731	-0.791
**29**	$C_{67}H_{125}N_{15}O_{13}$	1348.9654	1348.9650	1348.9650	-0.323	-0.315
**30**	$C_{66}H_{123}N_{15}O_{14}$	1350.9447	1350.9446	1350.9450	-0.037	0.244
**27**	$C_{70}H_{127}N_{13}O_{13}$	1358.9749	1358.9744	1358.9742	-0.359	-0.506
**28**	$C_{69}H_{125}N_{13}O_{14}$	1360.9542	1360.9538	1360.9533	-0.273	-0.670
**31**	$C_{69}H_{129}N_{15}O_{14}$	1392.9916	1392.9912	1392.9911	-0.323	-0.388
-	$C_{69}H_{127}N_{15}O_{14}$	1390.976	1390.9767	1390.9754	0.539	-0.410
**32**	$C_{72}H_{131}N_{13}O_{14}$	1403.0011	1403.0007	1403.0006	-0.322	-0.401

Figure S29: MALDI–HRMS spectra of KK7.4 wild type (WT) and pCEP_*fcl* promoter-exchange mutant (induced and noninduced) with compounds **2** and **27**–**32** showing sum formulas, calculated and detected masses and corresponding Δppm.

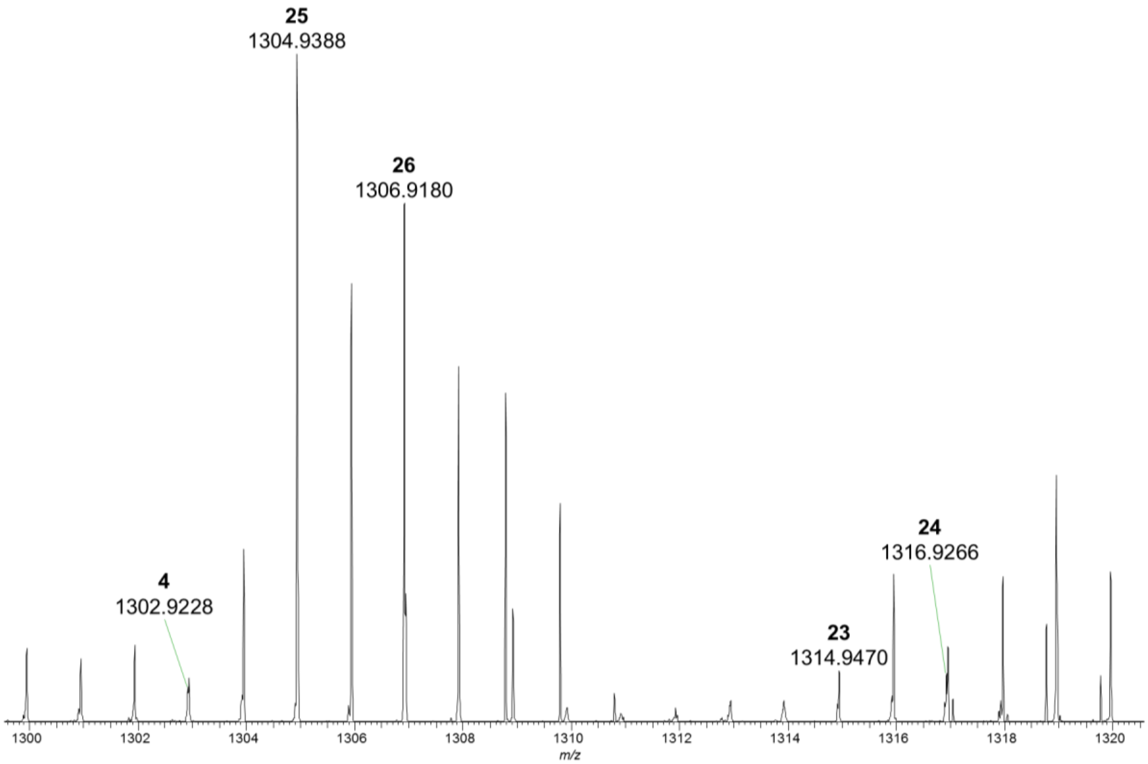

#	Sum formula	Calc. m/z	Det. m/z	Δppm
**4**	$C_{65}H_{119}N_{15}O_{12}$	1302.9235	1302.9228	-0.607
**25**	$C_{65}H_{121}N_{15}O_{12}$	1304.9392	1304.9388	-0.299
**26**	$C_{64}H_{119}N_{15}O_{13}$	1306.9185	1306.9180	-0.356
**23**	$C_{68}H_{123}N_{13}O_{12}$	1314.9487	1314.9470	-1.280
**24**	$C_{67}H_{121}N_{13}O_{13}$	1316.928	1316.9266	-1.015

Figure S30: MALDI–HRMS spectra of *X. innexi* wild type with compounds **4** and **23–26** showing sum formulas, calculated and detected masses and corresponding Δppm.

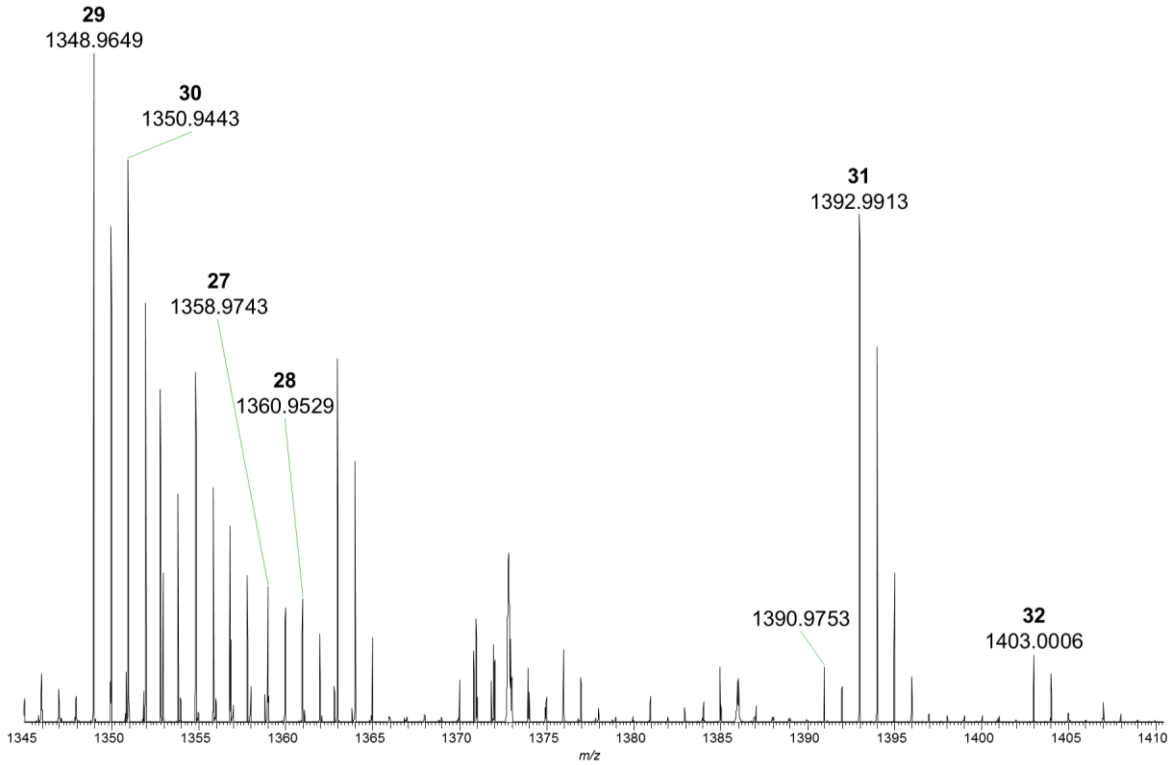

#	Sum formula	Calc. m/z	Det. m/z	∆ppm
**29**	$C_{67}H_{125}N_{15}O_{13}$	1348.9654	1348.9649	-0.374
**30**	$C_{66}H_{123}N_{15}O_{14}$	1350.9447	1350.9443	-0.281
**27**	$C_{70}H_{127}N_{13}O_{13}$	1358.9749	1358.9743	-0.462
**28**	$C_{69}H_{125}N_{13}O_{14}$	1360.9542	1360.9529	-0.912
-	$C_{69}H_{127}N_{15}O_{14}$	1390.976	1390.9753	-0.460
**31**	$C_{69}H_{129}N_{15}O_{14}$	1392.9916	1392.9913	-0.244
**32**	$C_{72}H_{131}N_{13}O_{14}$	1403.0011	1403.0006	-0.351

Figure S31: MALDI–HRMS spectra of *X. innexi* wild type with compounds **27**–**32** showing sum formulas, calculated and detected masses and corresponding ∆ppm.

Expected Fragmentions	Sum formula	m/z
y1	$C_{36}H_{80}N_5O$	598.6357
y2	$C_{47}H_{101}N_6O_5$	829.7828
y5	$C_{59}H_{118}N_{11}O_{10}$	1140.9058
y6	$C_{65}H_{125}N_{14}O_{11}$	1277.9647
b1	$C_4H_6NO_3$	116.0342
b2	$C_{10}H_{13}N_4O_4$	253.0931
b5	$C_{22}H_{30}N_9O_9$	564.2161
b6	$C_{33}H_{51}N_{10}O_{13}$	795.3632

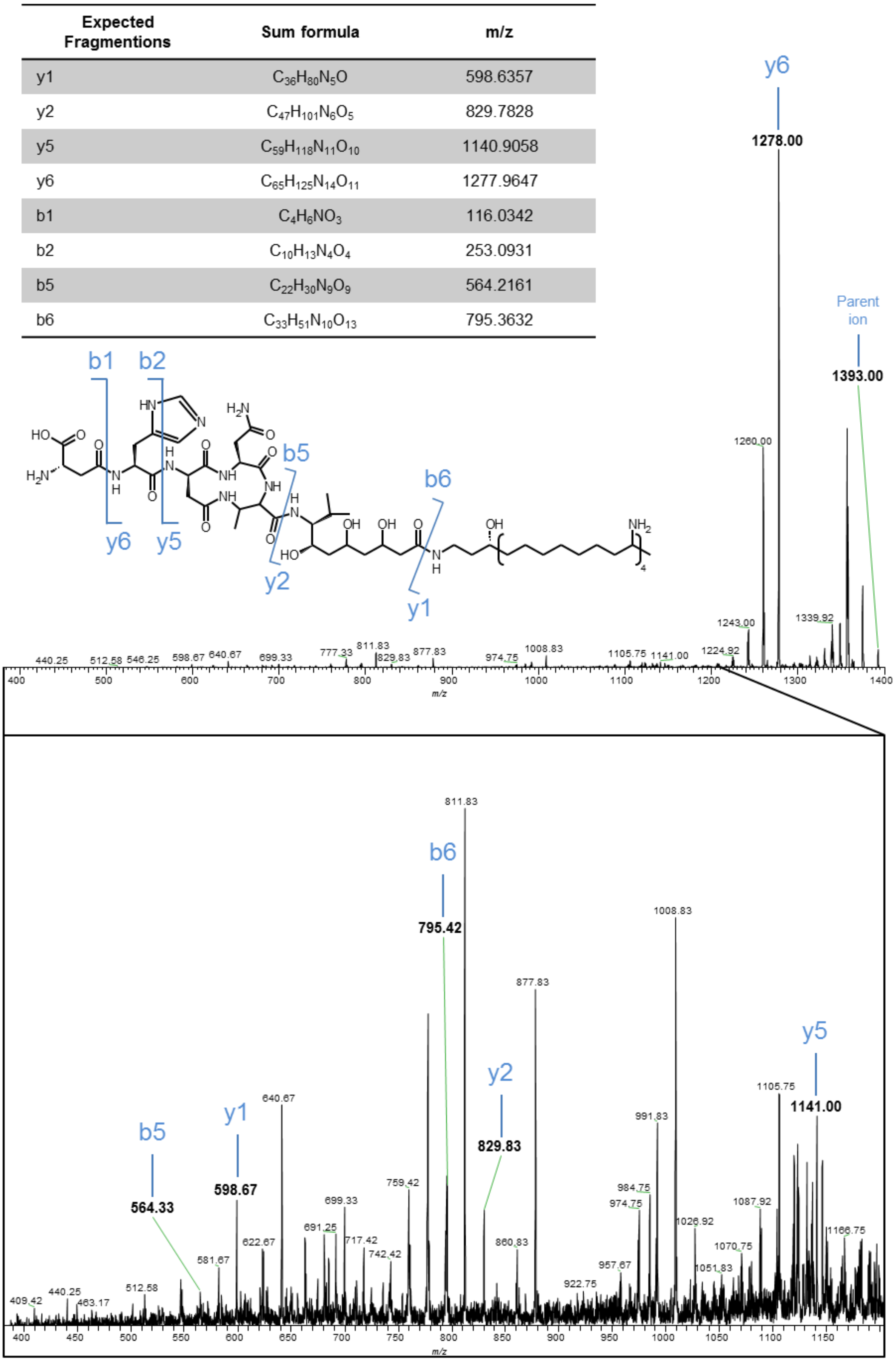

Figure S32: MALDI–MS² spectra of compound 1392.99 (**31**) of *X. innexi* showing expected fragment ions and proposed structure.

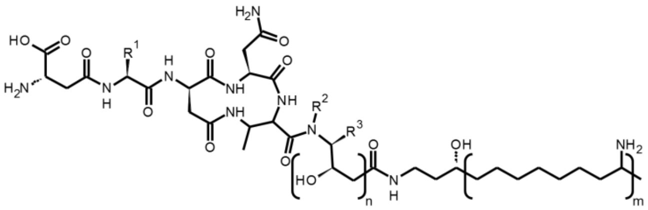

Occurence	R^1	R^2	R^3	n	m	Sum formula	$[M+H]^+$
a	CH_3	H	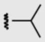	2	3	$C_{56}H_{106}N_{12}O_{13}$	1155.8075
a	CH_3	H	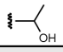	2	3	$C_{55}H_{104}N_{12}O_{14}$	1157.7868
a	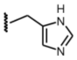	H	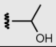	2	3	$C_{58}H_{106}N_{14}O_{14}$	1223.8086
b		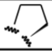		3	4	$C_{69}H_{127}N_{15}O_{14}$	1390.9760
a	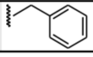	H	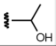	2	3	$C_{61}H_{108}N_{12}O_{14}$	1233.8181

Figure S33: Proposed minor derivatives found in *Xenorhabdus* strains (a: *X. szentirmaii*; b: KJ12.1, KK7.4). Identification is based on MALDI–HRMS spectra (Figure S2, S3, S22, and S29).

References

1. Wenski, S. L.; Kolbert, D.; Grammbitter, G. L. C.; Bode, H. B. *J. Ind. Microbiol. Biotechnol.* **2019**, 46 (3-4), 565-572. doi:10.1007/s10295-018-02124-8
2. Bode, E.; Brachmann, A. O.; Kegler, C.; Simsek, R.; Dauth, C.; Zhou, Q.; Kaiser, M.; Klemmt, P.; Bode, H. B. *Chembiochem* **2015,** *16* (7), 1115–1119. doi:10.1002/cbic.201500094
3. Simon, R.; Priefer, U.; Pühler, A. *Nat Biotechnol* **1983,** *1*, 784 EP -. doi:10.1038/nbt1183-784
4. Thoma, S.; Schobert, M. *FEMS Microbiol. Lett.* **2009,** *294* (2), 127–132. doi:10.1111/j.1574-6968.2009.01556.x
5. Fu, C.; Donovan, W. P.; Shikapwashya-Hasser, O.; Ye, X.; Cole, R. H. *PLoS ONE* **2014,** *9* (12), e115318. doi:10.1371/journal.pone.0115318
6. Agarwal, P.; Agarwal, N.; Gupta, R.; Gupta, M.; Sharma, B. *J Microb Biochem Technol* **2016,** *08* (05). doi:10.4172/1948-5948.1000316
7. Fang, X. L.; Li, Z. Z.; Wang, Y. H.; Zhang, X. *J. Appl. Microbiol.* **2011,** *111* (1), 145–154. doi:10.1111/j.1365-2672.2011.05033.x
8. Lengyel, K.; Lang, E.; Fodor, A.; Szállás, E.; Schumann, P.; Stackebrandt, E. *Syst. Appl. Microbiol.* **2005,** *28* (2), 115–122. doi:10.1016/j.syapm.2004.10.004
9. Tailliez, P.; Pagès, S.; Ginibre, N.; Boemare, N. *Int. J. Syst. Evol. Microbiol.* **2006,** *56* (Pt 12), 2805–2818. doi:10.1099/ijs.0.64287-0
10. Somvanshi, V. S.; Lang, E.; Ganguly, S.; Swiderski, J.; Saxena, A. K.; Stackebrandt, E. *Syst. Appl. Microbiol.* **2006,** *29* (7), 519–525. doi:10.1016/j.syapm.2006.01.004
11. Thanwisai, A.; Tandhavanant, S.; Saiprom, N.; Waterfield, N. R.; Ke Long, P.; Bode, H. B.; Peacock, S. J.; Chantratita, N. *PLoS ONE* **2012,** *7* (9), e43835. doi:10.1371/journal.pone.0043835
12. Tobias, N. J.; Wolff, H.; Djahanschiri, B.; Grundmann, F.; Kronenwerth, M.; Shi, Y.-M.; Simonyi, S.; Grün, P.; Shapiro-Ilan, D.; Pidot, S. J.; Stinear, T. P.; Ebersberger, I.; Bode, H. B. *Nat. Microbiol.* **2017,** *2* (12), 1676–1685. doi:10.1038/s41564-017-0039-9
13. Hurst, S. G.; Ghazal, S.; Morris, K.; Abebe-Akele, F.; Thomas, W. K.; Badr, U. M.; Hussein, M. A.; AbouZaied, M. A.; Khalil, K. M.; Tisa, L. S. *Genome Announc* **2014,** *2* (6). doi:10.1128/genomeA.01273-14
14. Donmez Ozkan, H.; Cimen, H.; Ulug, D.; Wenski, S.; Yigit Ozer, S.; Telli, M.; Aydin, N.; Bode, H. B.; Hazir, S. *Front Microbiol* **2019,** *10*, 2672. doi:10.3389/fmicb.2019.02672
15. Stachelhaus, T.; Mootz, H. D.; Marahiel, M. A. *Chem. Biol.* **1999,** 6 (8), 493-505. doi: 10.1016/S1074-5521(99)80082-9
16. Challis, G. L.; Ravel, J.; Townsend, C. A. *Chem. Biol.* **2000,** 7 (3), 211-224. doi: 10.1016/s1074-5521(00)00091-017.Kim, I.-H.; Aryal, S. K.; Aghai, D. T.; Casanova-Torres, Á. M.; Hillman, K.; Kozuch, M. P.; Mans, E. J.; Mauer, T. J.; Ogier, J.-C.; Ensign, J. C.; Gaudriault, S.; Goodman, W. G.; Goodrich-Blair, H.; Dillman, A. R. *BMC Genomics* **2017,** *18* (1), 927. doi:10.1186/s12864-017-4311-4
17. Kim, I.-H.; Aryal, S. K.; Aghai, D. T.; Casanova-Torres, Á. M.; Hillman, K.; Kozuch, M. P.; Mans, E. J.; Mauer, T. J.; Ogier, J.-C.; Ensign, J. C.; Gaudriault, S.; Goodman, W. G.; Goodrich-Blair, H.; Dillman, A. R. *BMC Genomics* **2017,** *18* (1), 927. doi:10.1186/s12864-017-4311-4
18. Masschelein, J.; Mattheus, W.; Gao, L.-J.; Moons, P.; van Houdt, R.; Uytterhoeven, B.; Lamberigts, C.; Lescrinier, E.; Rozenski, J.; Herdewijn, P.; Aertsen, A.; Michiels, C.; Lavigne, R. *PLoS ONE* **2013,** *8* (1), e54143. doi:10.1371/journal.pone.0054143

19. Masschelein, J.; Clauwers, C.; Awodi, U. R.; Stalmans, K.; Vermaelen, W.; Lescrinier, E.; Aertsen, A.; Michiels, C.; Challis, G. L.; Lavigne, R. *Chem. Sci.* **2015,** *6* (2), 923–929. doi:10.1039/c4sc01927j
20. Fuchs, S. W.; Grundmann, F.; Kurz, M.; Kaiser, M.; Bode, H. B. *Chembiochem* **2014,** *15* (4), 512–516. doi:10.1002/cbic.201300802
